# Potential of Central, Eastern and Western Africa Medicinal Plants for Cancer Therapy: Spotlight on Resistant Cells and Molecular Targets

**DOI:** 10.3389/fphar.2017.00343

**Published:** 2017-06-02

**Authors:** Armelle T. Mbaveng, Victor Kuete, Thomas Efferth

**Affiliations:** ^1^Department of Pharmaceutical Biology, Institute of Pharmacy and Biochemistry, University of MainzMainz, Germany; ^2^Department of Biochemistry, Faculty of Science, University of DschangDschang, Cameroon

**Keywords:** Africa, cancer, plants, phytochemicals, molecular targets, resistance

## Abstract

Cancer remains a major health hurdle worldwide and has moved from the third leading cause of death in the year 1990 to second place after cardiovascular disease since 2013. Chemotherapy is one of the most widely used treatment modes; however, its efficiency is limited due to the resistance of cancer cells to cytotoxic agents. The present overview deals with the potential of the flora of Central, Eastern and Western African (CEWA) regions as resource for anticancer drug discovery. It also reviews the molecular targets of phytochemicals of these plants such as ABC transporters, namely P-glycoprotein (P-gp), multi drug-resistance-related proteins (MRPs), breast cancer resistance protein (BCRP, ABCG2) as well as the epidermal growth factor receptor (EGFR/ErbB-1/HER1), human tumor suppressor protein p53, caspases, mitochondria, angiogenesis, and components of MAP kinase signaling pathways. Plants with the ability to preferentially kills resistant cancer cells were also reported. Data compiled in the present document were retrieved from scientific websites such as PubMed, Scopus, Sciencedirect, Web-of-Science, and Scholar Google. In summary, plant extracts from CEWA and isolated compounds thereof exert cytotoxic effects by several modes of action including caspases activation, alteration of mitochondrial membrane potential (MMP), induction of reactive oxygen species (ROS) in cancer cells and inhibition of angiogenesis. Ten strongest cytotoxic plants from CEWA recorded following *in vitro* screening assays are: *Beilschmiedia acuta* Kosterm, *Echinops giganteus* var. lelyi (C. D. Adams) A. Rich., *Erythrina sigmoidea* Hua (Fabaceae), *Imperata cylindrical* Beauv. var. koenigii Durand et Schinz, *Nauclea pobeguinii* (Pobég. ex Pellegr.) Merr. ex E.M.A., *Piper capense* L.f., *Polyscias fulva* (Hiern) Harms., *Uapaca togoensis* Pax., *Vepris soyauxii* Engl. and *Xylopia aethiopica* (Dunal) A. Rich. Prominent antiproliferative compounds include: isoquinoline alkaloid isotetrandrine (**51**), two benzophenones: guttiferone E (**26**) and isoxanthochymol (**30**), the isoflavonoid 6α-hydroxyphaseollidin (**9**), the naphthyl butenone guieranone A (**25**), two naphthoquinones: 2-acetylfuro-1,4-naphthoquinone (**4**) and plumbagin (**37**) and xanthone V_1_ (**46**). However, only few research activities in the African continent focus on cytotoxic drug discovery from botanicals. The present review is expected to stimulate further scientific efforts to better valorize the African flora.

## Introduction

Cancer is a term for a series of malign diseases characterized by abnormal cell proliferation, leading to invasion and metastasis, the ultimate causes of deaths by cancer. The burden of neoplastic diseases affects the entire world population. Over the past two decades, there has been a slight improvement in cancer statistics due to diagnostic and therapeutic progresses and a better understanding of tumor biology (Siegel et al., 2014). However, cancer remains associated with very high mortality rates, which indicate still existing difficulties of effective treatment. Chemotherapy is one of the most widely used modes of anti-cancer therapy. However, the development of resistance of cancer cells to cytotoxic agents represents a main factor, which is responsible for the non-satisfactory treatment outcomes associated with malignant diseases (Singh and Settleman, 2010). In fact, most types of cancer cells reveal variable degrees of resistance to antineoplastic agents (Luqmani, 2005). In 2008, men in Africa had more than double of the rate of world liver cancer cases, whilst women had the highest incidence of cervical cancer of the world. Medicinal plants have long been used to fight against cancer. Several natural products isolated from medicinal plants including: terpenoids, phenolics, and alkaloids play an important role in cancer treatment (Kaur et al., 2011). More than 3,000 plants worldwide have been reported to exert cytotoxicity toward cancer cells (Graham et al., 2000; Solowey et al., 2014). About 80% of the rural African population almost exclusively uses traditional medicine for its primary health care needs (Farnsworth et al., 1985). For cultural and economic reasons, medicinal plants constitute the major part of traditional medicine. In the recent years, numerous African medicinal plants have been screened for their cytotoxic potential. This review deals with plants and derived molecules from Central, Eastern and Western Africa (CEWA) as potential resource for cancer chemotherapy with emphasis on their molecular targets. Countries of Central Africa include: Cameroon, Gabon, Equatorial Guinea, Central African Republic, Congo, Democratic Republic of Congo, São Tomé and Príncipe, Chad, Angola. East Africa comprises of Kenya, Uganda, Tanzania, Rwanda, Burundi, Sudan, Eritrea, Djibouti, Ethiopia, Somalia, Seychelles, Comoros, Mauritius Island, Madacascar, Mozambique, and Malawi. Western African countries include Benin, Burkina Faso, Ivory Coast, Gambia, Ghana, Guinea, Guinea- Bissau, Cape Verde, Nigeria, Mali, Mauritania, Niger, Liberia, Senegal, Sierra Leone, and Togo. Hence, the medicinal plants of CEWA described in the present review cover a considerable portion of the African continent.

## Overview of cancer burdenin africa

Cancer moved from the third leading cause of death worldwide in 1990 to the second leading cause of death after cardiovascular disease since 2013, with more than 8 million deaths in 2013 (Murray and Lopez, 1997; Lozano et al., 2012). Although significant progress has been made in recent years in cancer prevention and treatment (Edwards et al., 2014; Allemani et al., 2015), the burden of cancer is increasing as a result of a growing and aging population worldwide, in addition to risk factors such as smoking, obesity and diet. To adequately allocate resources for prevention, screening, diagnosis, treatment and palliative care, and to monitor its effectiveness, there is an urgent need for timely information on the burden of cancer for each country. It is worth noting that in several African countries, the cancer burden still remains unclear in terms of reliable epidemiological data, though most practicing physicians recognize that the number of cases among patients visiting local health facilities continuously increases (Omosa et al., 2015). By 2020, 15 million new cancer cases are annually expected, 70% of which will be from developing countries. African countries will account for more than a million new cancer cases per year and have to cope with them despite few cancer care services (Vorobiof and Abratt, 2007). In Africa, about a third of cancer deaths are potentially preventable. In sub-Saharan Africa in 2002, more than half a million deaths from cancer were reported, with nearly 40% related to chronic infections and smoking (Vorobiof and Abratt, 2007). Due to the lack of basic resources and infrastructure, most Africans, including those in CEWA, do not have access to cancer screening, early diagnosis, appropriate treatment or palliative care. For example, radiotherapy is available in only 21 of the 53 African countries, reaching less than 5% of the population, and consequently patients are deprived of life-saving treatment (Vorobiof and Abratt, 2007).

## Molecular targets of phytochemicals and their role in the resistance of tumors to cytotoxic drugs

The role of phytochemicals as cytotoxic agents against cancer cell lines has frequently been reported. Various plant molecules including nutraceuticals, such as allicin, apigenin, berberine, catechin gallate, celastrol, curcumin, epigallocatechin gallate, fisetin, flavopiridol, gambogicacid, genistein, plumbagin, quercetin, resveratrol, silibinin, taxol, etc. derived from spices, legumes, fruits, nuts, and vegetables have been shown to modulate inflammatory pathways and exert inhibitory effects against tumor cells (Chirumbolo, 2012). Several other molecules from medicinal plants are already clinically established for cancer treatment, for example alkaloids such as vinblastine and vincristine isolated from *Catharanthus roseus* (Gullett et al., 2010), combretastatins isolated from *Combretum caffrum* (Cirla and Mann, 2003), paclitaxel, obtained from *Taxus brevifolia* (Luduena, 1998), camptothecin isolated from *Camptotheca acuminata* and homoharringtonine isolated from *Cephalotaxus harringtonia* (Aboul-Enein et al., 2014). Phytochemicals and nutraceuticals have frequently many molecular targets. Targets of natural products include: Aurora-A, Cdc2, Cdc25a, Cyclin B1, Cyclin D1, E2F4, RB, FoxM1, Skp2, p16, p21, p27 (cell cycle), EGFR, IGF-I, IGF-II, IGF-1R, IGFBP-1, IGFBP-2, IGFBP-3, IGFBP-5, ERK, JNK/c-Jun, p38, Akt, mTOR, PI3K, PTEN, 4E-BP1, G3BP1, Ras, ErbB2 (growth factor signaling), androgen receptor, estrogen receptors (ERα, ERβ), (hormone signaling), FOXO, C/EBPα, BTG3, PHB, Pin1, PKCα, PKCδ, RARα, RARβ, VDR, telomerase (Non-classified targets). Targets involving apoptotic pathways include Apaf-1, GDF15, BAD, Bax, Bcl-2, Bcl-xL, Bcl-xS, caspases 3, 8, 9, and 10, cIAP1, XIAP, DR5, Fas, Hsp70 andsurvivin. Phytochemicals are also involved in other cell activities such as cell metabolism modification (SphK1, HIF-1α, FASN, HMG-CoA reductase, AMPK, PFKFB4), drug resistance inhibition (MRP5, BCRP, P-glycoprotein), genome stability (ATM/Chk1, BRCA1, BRCA2, p53, topoisomerase-II), inhibition of immune evasion (IL-10, IDO, TGFβ), inhibition of invasion, metastasis and angiogenesis (E-cadherin, CXCL1, CXCL2, CXCL12, CXCR4, EMMPRIN, connexin 43, KAI1, c-Met, endoglin, VEGF/VEGFR, vimentin, ZEB1, MMP-2, -7, -9, PAK1) and stemness inhibition (Gli1, WIF-1, Wnt/β-catenin, Notch-1, Notch-2, Twist-1), antioxidant/carcinogen metabolism (hSULT1A1, hSULT2A1, UGT1A, QR, GST, Nrf2, ARE, CYP1A1, metallothionein), anti-inflammation (IL-1RI, CCL2, NF-κB, IKK, COX-1, COX-2, PGE2, iNOS, PPARγ) (Gonzalez-Vallinas et al., 2013). However, in this section, we will discuss the most currently investigated targets of plants and their derived molecules as well as those involved in cancer drug resistance.

### ABC transporters and drug resistance

The adenosine triphosphate (ATP)-binding cassette (ABC) proteins are amongst the largest protein families found in all living organisms from microbes to humans (Efferth and Volm, 2017). The roles of ABC transporters include binding to and hydrolysis of ATP to fuel energy-dependent efflux of specific compounds across the membrane or to return them from the inner to the outer surface of membranes (Dean, 2009). Malignant cells resist to anticancer drugs by mutation or overexpression of drug targets, as well as by inactivation or efflux of the compounds to prevent cytotoxic drug concentrations sufficient to kill tumor cells (Gottesman et al., 2006). Human ABC transporters involved in drug resistance include ABCA3 or ABC3/ABCC (ABCA family), ABCB1 or MDR1/P-glycoprotein (P-gp) (ABCB family), ABCC1 or MRP1 and ABCC3 or MRP3/cMOAT-2 (ABCC family), ABCG2 or ABCP/MXR/BCRP (ABCG family) (Glavinas et al., 2004). The roles of P-gp, multidrug-resistance-proteins (MRPs) and breast cancer resistance protein (BCRP) in cancer drug resistance have been intensively investigated (Efferth, 2001; Gillet et al., 2007).

#### P-glycoprotein (P-gp)

P-gp is encoded by the *ABCB1/MDR1* gene and was identified as the first ABC transporter to be overexpressed in multidrug resistant cancer cell lines (Kartner et al., 1985). P-gptransports and/or secretes substrates in normal tissues such as the kidney, liver, colon, and adrenal gland as well as in the blood-brain, blood-placenta, and blood-testis barriers to protect these tissues from harmful compounds (Katayama et al., 2014). P-gp is involved in the efflux of doxorubicin, daunorubicin, vincristine, etoposide, colchicine, camptothecins and methotrexate, leading to resistance of cancer cells to these molecules (Dean, 2009). Clinical trials with synthetic drugs undertaken since 1994 have not resulted in significant progress in the discovery of new blockbusters for chemotherapy (Dean et al., 2005). Combating cancer-drug-resistance with phytochemicals inhibiting *ABCB1* could therefore be a more promising strategy to overcome multi-drug resistance (MDR). Additionally, other ABC transporters such as ABCC1/MRP1 (Cole et al., 1992) and ABCG2 (Kim et al., 2002) are also overexpressed in cancer cells and could be targeted by plant products.

#### Multidrug-resistance-related proteins (MRPs)

MRPs comprise of at least 9 types of transporters termed MRP1-9. They transport a wide array of structurally diverse molecules across cell membranes. They are involved in the absorption, disposition, and elimination of compounds in the body (Tian et al., 2005). MRPs are ATP-dependent efflux pumps having broad substrate specificity for the transport of endogenous substances such as glutathione conjugates (leukotriene C4 for MRP1, MRP2, and MRP4), bilirubin glucuronosides (MRP2 and MRP3), and cyclic AMP and cyclic GMP (MRP4, MRP5, and MRP8) as well as xenobiotic anionic substances localized in cellular plasma membranes (Keppler, 2011). Their overexpression in malignant cells is associated with resistance to a number of important cytotoxic drugs. MRPs are involved in the efflux of several anticancer drugs such as doxorubicin, daunorubicin, vincristine, etoposide, colchicine, camptothecins, methotrexate (MRP1), vinblastine, cisplatin, doxorubicin, methotrexate (MRP2), methotrexate, etoposide (MRP3), 6-mercaptopurine (6-MP), 6-thioguanine (6-TG), methotrexate (MRP4), 6-MP and 6-TG (MRP5), etoposide (MRP6) and 5-fluorouracil (MRP8) (Dean, 2009).

#### Breast cancer resistance protein (BCRP, ABCG2)

MXR alias BCRP is an ABC transporter that plays a role in absorption, distribution, metabolism and excretion in normal tissues (Natarajan et al., 2012). Its overexpression in tumor cells confers resistance to chemotherapy by active extrusion of cytotoxic compounds. BCRP is involved in the efflux of mitoxantrone, topotecan, doxorubicin, daunorubicin, irinotecan, imatinib, and methotrexate (Dean, 2009). This receptor protein is involved in MDR of several tumor types including: acute leukemia and other hematological malignancies, head and neck carcinoma, breast cancer, lung cancer, brain tumors, hepatocellular carcinoma, gastrointestinal cancers such as pancreatic, colon, gastric and esophageal carcinomas (Natarajan et al., 2012).

### Epidermal growth factor receptor (EGFR/ErbB-1/HER1)

The epidermal growth factor receptor (EGFR; ErbB-1; HER1), a signal transducer for cell growth and differentiation, is the cell-surface receptor belonging to the ErbB family of receptors. This family consists of four closely related receptor tyrosine kinases, namely EGFR/HER1/ErbB-1, HER2/c-neu/ErbB-2, HER3/ErbB-3, and HER4/ErbB-4. Mutations affecting the activity or expression of EGFR can contribute to carcinogenesis (Zhang et al., 2007). Upon stimulation by ligands, EGFR is activated through homodimerization or heterodimerization and transmit signals to downstream substrates such as PI3K/AKT, RAS/RAF/MAPK, and STAT3/5 pathways, leading to cell proliferation and cell survival (Ji, 2010). Downstream substrates of EGFR have been found responsible for drug resistance meanwhile activation of PI3K/AKT pathway is essential for cancer cell survival (Ji, 2010). ErbB family receptors represent important targets of anticancer therapeutics such as tyrosine kinase inhibitors (TKIs; for example gefitinib and erlotinib) (Zhang et al., 2007).

### Human tumor suppressor protein p53

The tumor suppressor protein *p53* is encoded by the *TP53* gene in human beings and *Trp53* gene in mice. It is crucial in multicellular organisms, where it prevents cancer formation, thus, functions as a tumor suppressor (Surget et al., 2013). The gene *p53* is involved in the regulation of cell fate in response to different stresses in normal cells through the differential regulation of gene expression. Abnormal *p53* expression actively contributes to cancer formation and progression in malignant cells. The gene *p53* is also associated with response to cancer treatment by regulating apoptosis, genomic stability, and angiogenesis. Overexpression of mutated *p53* with reduced or abolished function is often associated with resistance to various cytotoxic drug such as cisplatin, temozolomide, doxorubicin, gemcitabine, tamoxifen, and cetuximab (Hientz et al., 2017).

### Caspases as anticancer drug target

Caspases or cysteine-aspartic proteases are a family of protease enzymes essential for programmed cell death and inflammation. There are 14 mammalian caspases, 12 of which are of human origin (caspases 1–10, 12, and 14). They can be classified into three main types, that are initiator caspase (2, 8, 9, and 10), executioner or effector caspases (3, 6, and 7) and inflammatory caspases (1, 4, 5, 11, and 12) (Galluzzi et al., 2016). Caspase-14 plays a role in epithelial cell keratinocyte differentiation, and forms an epidermal barrier that protects against dehydration and UVB radiation (Denecker et al., 2008). Upon activation, caspases cleave a variety of substrates including: proteins involved in signal transduction (apoptosis regulators, cytokines, serine/threonine kinases), structural proteins (cytoskeletal and nuclear) and proteins involved in regulation of transcription, translation and RNA editing (Howley and Fearnhead, 2008). Deregulation of caspase activation or expression also leads to neurodegenerative and autoinflammatory disorders (Howley and Fearnhead, 2008). Initiator caspase-9 is activated in the apoptosome, while caspase-2 is activated in the PIDDosome and caspase-8 or -10 in the death-inducing signaling complex (DISC) (Howley and Fearnhead, 2008). Activated initiator caspases activate effector caspases, which in turn cleave structural and regulatory proteins culminating in the features of apoptosis. The search for caspase modulators is a novel attractive therapeutic approach in cancer research (Howley and Fearnhead, 2008).

### Mitochondria as anticancer drug target

Mitochondria play a central role in cellular metabolism, calcium homeostasis, redox signaling, and cell fate as main ATP source. During ATP biosynthesis, reactive oxygen species (ROS) are generated. In many cancer cells, mitochondria appear to be dysfunctional (due to a variety of factors, such as oncogenic signals and mitochondrial DNA mutations), with a shift in energy metabolism from oxidative phosphorylation to active glycolysis and an increase in the generation of ROS (Wen et al., 2013). The energy metabolism is different between normal and cancer cells, providing a scientific basis for development of strategies to selectively target malignant cells. As a result of mitochondrial dysfunction, cancer cells rely more on the glycolytic pathway in the cytosol to generate ATP. Key enzymes in this pathway such as hexokinase II, glyceraldehyde 3-phosphate dehydrogenase (overexpressed in malignant cells) therefore became potential therapeutic targets (Wen et al., 2013). Mitochondria-targeting compounds can kill drug-resistant cancer cells due to their ability to initiate mitochondrial outer membrane permeabilization in mitochondria, independently of other upstream signaling processes that may be impaired in cancer cells (Fulda and Kroemer, 2011). Some potential therapeutic targets associated with mitochondria include NADPH oxidases (NOX), the translocator protein (TSPO), the mitochondrial protein known as complement component 1, q subcomponent-binding protein (C1qBP) and the monocarboxylate transporters (MCTs) (Wen et al., 2013). Compounds known to target the mitochondrial membrane potential are for instance the natural alkaloid pancratistatin, rhodamine-123, 4-phenyl-2,7-di(piperazin-1-yl)-1,8-naphthyridine, 2,5-diaziridinyl-3- (hydroxymethyl)-6-methyl-1,4-benzoquinone and edelfosine (Wen et al., 2013). Natural products such ascurcumin, resveratrol, berberine and cerulenin target mitochondrial apoptotic pathway (Wen et al., 2013).

### Reactive oxygen species and cancer chemotherapy

Reactive oxygen species are chemically reactive chemical species containing oxygen such as hydroxyl radical, peroxides, superoxide, and singlet oxygen. They are produced through multiple mechanisms depending on cell and tissue types by NOX complexes in cell membranes, mitochondria, peroxisomes and endoplasmic reticulum (Muller, 2000; Han et al., 2001). ROS not only induce apoptosis, but also regulate host defense genes or airway homeostasis (Conner et al., 2002; Rada and Leto, 2008). In malignant cells, ROS induce changes in cellular functions such as cell death, cell proliferation, migration and differentiation (Wen et al., 2013). Increased ROS levels and mitochondrial dysfunction make cancer cells more vulnerable than normal cells.

### Angiogenesis as anticancer drug target

Angiogenesis is a physiological process in embryogenesis, in wound healing and in the female reproductive cycle leading to the formation of new blood vessels from pre-existing ones (Kumaran et al., 2008; Birbrair et al., 2015). Angiogenesis is critical in cancer for growth and metastasis, as tumors cannot grow beyond 200–300 μm in diameter without recruitment of new blood vessels to maintain nutrients and oxygen supply (Kumaran et al., 2008). This also makes angiogenesis an ideal target for cancer treatment. Some established therapeutic strategies targeting angiogenesis include bevacizumab [antibody to vascular endothelial growth factor (VEGF)], sorafenib and sunitinib (tyrosine kinase inhibitors). Combretastatin (vascular disruptive agents) and endostatin (endogenous inhibitor) are currently in clinical trials (Kumaran et al., 2008).

### MAP kinase signaling pathways in cancer chemotherapy

The mitogen-activated protein kinases/extracellular signal-regulated kinases (MAPK/ERK) pathway or Ras-Raf-MEK-ERK pathway is one of the most important signal transduction pathways. The MAPK/ERK pathway regulates growth, proliferation, differentiation and survival of the cells. Its deregulation is observed in various diseases such as cancer, degenerative syndromes, immunological and inflammatory diseases, making it an important drug target (Orton et al., 2005). The activation of a MAPK employs a core three-kinase cascade consisting of a MAPK kinase kinase (MAP3K or MAPKKK), which phosphorylates/activates another MAPK kinase (MAP2K, MEK, or MKK), which in turn phosphorylates and activates more MAPKs. Upon activation, MAPKs can phosphorylate a variety of intracellular targets such as cytoskeletal elements, nuclear pore proteins, membrane transporters, transcription factors, and other protein kinases (Avruch et al., 2001). Mutations in proteins of this pathway, for example in Ras and B-Raf lead to carcinogenesis. Compounds targeting MAPK pathways are therefore investigated as potential cancer drugs (Orton et al., 2005). In fact, the role of stress-activated pathways such as Jun N-terminal kinase and p38 in the prevention of malignant transformation has been shown (Dhillon et al., 2007).

## Central, eastern and western africa plants and derived molecules and their anticancer targets

**Figure 1:**
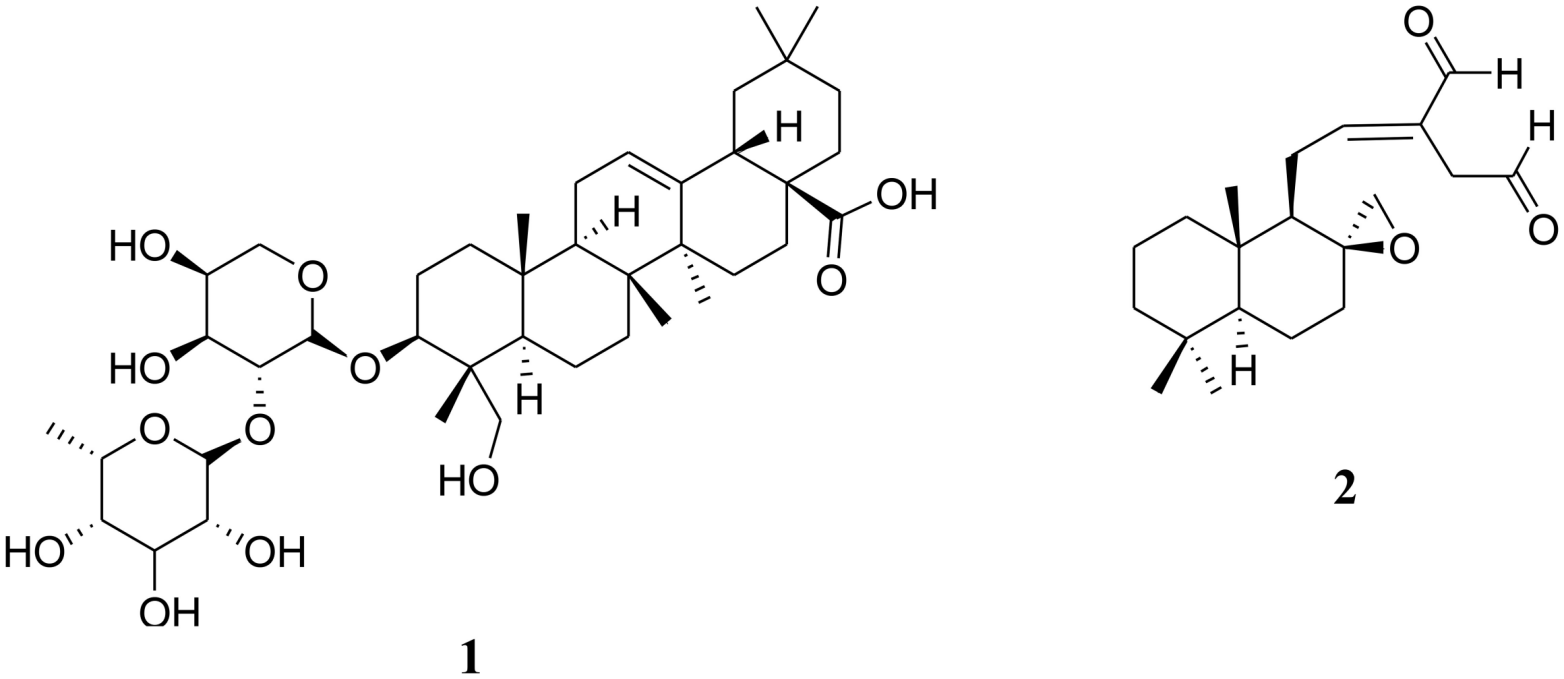
Chemical structures of two cytotoxic terpenoids [alpha-hederin (**1**) and galanal A (**2**)] isolated from Central, East and West African plants.

**Figure 2:**
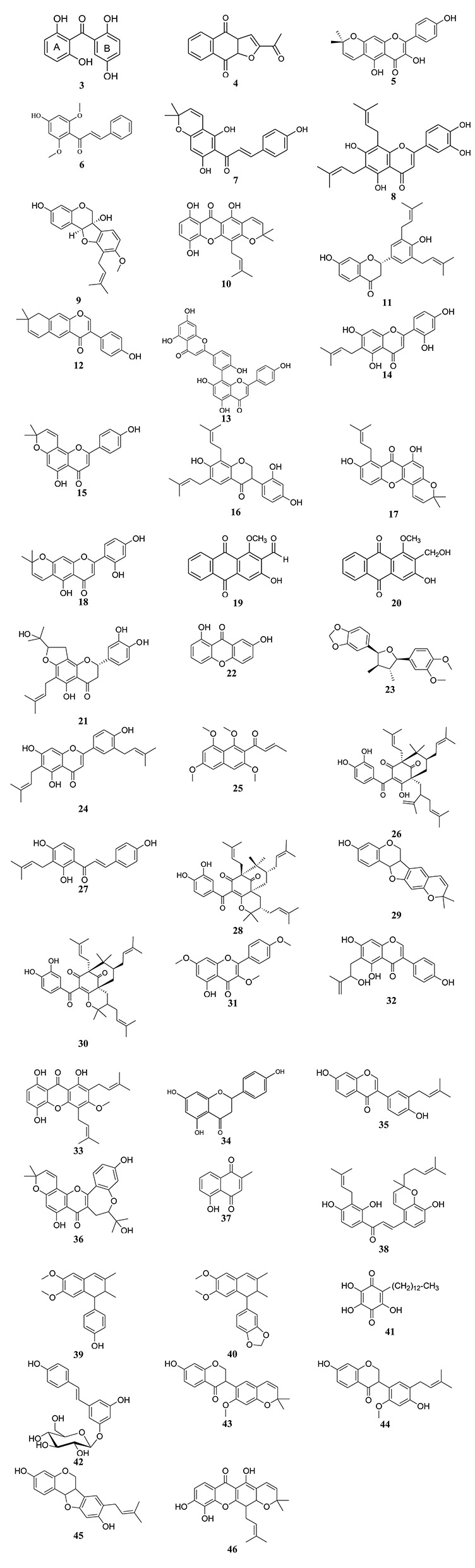
Chemical structures of hit cytotoxic phenolics isolated from Central, East and West African plants. 2,2′,5,6′-tetrahydroxybenzophenone 3); 2-acetylfuro-1,4-naphthoquinone (**4**); 3,4′,5-trihydroxy-6″,6″-dimethylpyrano[2,3-g]flavone (**5**); 4′-hydroxy-2′,6′-dimethoxychalcone (**6**); 4-hydroxylonchocarpin (**7**); 6,8-diprenyleriodictyol (**8**); 6α-hydroxyphaseollidin (**9**); 8-hydroxycudraxanthone G (**10**); abyssinone IV (**11**); alpinumisoflavone (**12**); amentoflavone (**13**); artocarpesin (**14**); atalantoflavone (**15**); bidwillon A (**16**); cudraxanthone I (**17**); cycloartocapesin (**18**); damnacanthal (**19**); damnacanthol (**20**); dorsmanin F (**21**); euxanthone (**22**); futokadsurin B (**23**); gancaonin Q (**24**); guieranone A (**25**); guttiferone E (**26**); isobavachalcone (**27**); isogarcinol (**28**); isoneorautenol (**29**); isoxanthochymol (**30**); kaempferol-3,7,4′-trimethylether (**31**); laburnetin (**32**); morusignin I (**33**); naringenin (**34**); neobavaisoflavone (**35**); neocyclomorusin (**36**); plumbagin (**37**); poinsettifolin B (**38**); pycnanthulignene A (**39**); pycnanthulignene B (**40**); rapanone (**41**); resveratrol β-*_D_*-glucopyranoside (**42**); sigmoidin H (**43**); sigmoidin I (**44**); sophorapterocarpan A (**45**); xanthone V_1_ (**46**).

**Figure 3:**
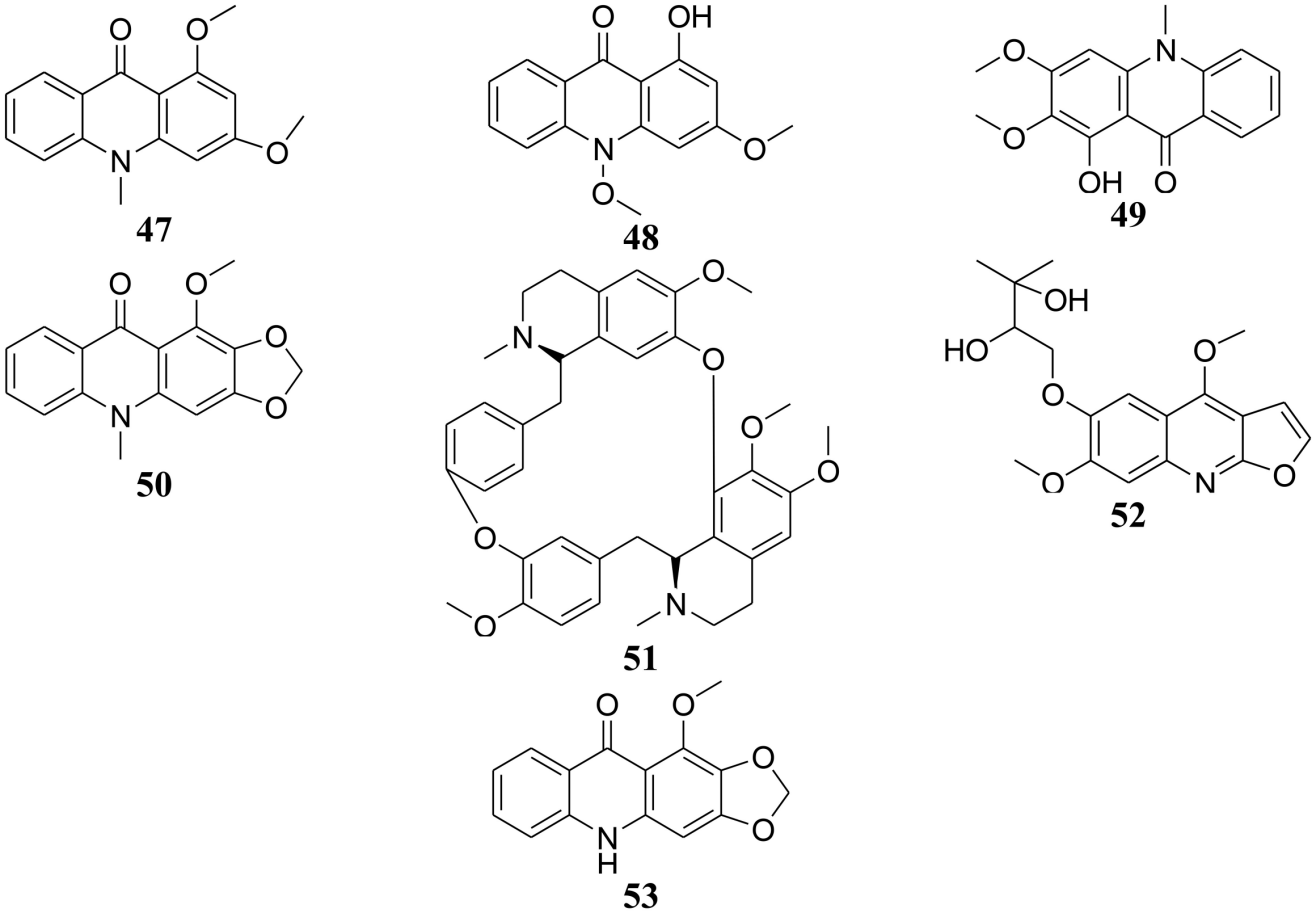
Chemical structures of hit cytotoxic alkaloids isolated from plants of Central, East and West Africa. 1,3-dimethoxy-10-methylacridone (**47**); 1-hydroxy-3-methoxy-10-methylacridone (**48**); arborinin (**49**); evoxanthine (**50**); isotetrandrine (**51**); montrifoline (**52**); norevoxanthine (**53**).

During the past decade, intensive investigations of African medicinal plants as potential anticancer drug candidates have been carried out by African scientists in collaboration with various research teams throughout the world. However, this work should be strenghtened with particular emphasis on the study of mechanisms of action and the identification of the different molecular targets of bioactive substances. Here, we give an overview of the studies published so far on plants and products derived from CEWA as far as their molecular target are available. A synopsis of phytochemicals acting preferentially on cancer cell lines actively expressing drug targets such ABC transporters, EGFR, p53 and BCRP (Figures 1–3) will also be given. For instance the degree of resistance (DR) determined as the ratio of IC_50_ value of the resistant/IC_50_ sensitive cell line will be taken into account to consider samples with potential therapeutic values to combat MDR phenotypes. Hence, samples with hypersensitivity or collateral sensitivity (more active on resistant than on parental sensitive cells line with DRs below 0.90 as well as samples with regular sensitivity (DR between 0.91 and 1.19) will be discussed. According to the criteria of the American National Cancer Institute, 20 μg/mL is the upper IC_50_ limit to be considered as promising for cytotoxic crude extracts (Suffness and Pezzuto, 1990). Meanwhile, a threshold of 4 μg/ml or 10 μM (Boik, 2001; Brahemi et al., 2010) after 48–72 h incubation has been set to identify compounds with considerable cytotoxic activity.

### Caspases activators

Although many African plant extracts were poor caspase activators (Kuete and Efferth, 2015), several phytochemicals from the flora of CEWA were reported as caspase activators (Tables 1, 2). Some documented caspase 3/7 activators included: benzophenones: guttiferone E (**26**) (Kuete et al., 2013d) and isoxanthochymol (**30**) from *Garcinia punctata* Oliv. (Kuete et al., 2013d), flavonoids: 4-hydroxylonchocarpin (**7**) and isobavachalcone (**27**) isolated from *Dorstenia barteri* Bureau (Kuete et al., 2011b, 2015c), 6,8-diprenyleriodictyol (**8**) isolated from *Dorstenia mannii* Hook.f. (Kuete et al., 2011b), cycloartocarpesin (**18**) from *Morus mesozygia* Stapf. (Kuete et al., 2015c), gancaonin Q (**24**) from *Dorstenia angusticornis* Engl. (Kuete et al., 2011b), isoflavonoids: 6α-hydroxyphaseollidin (**9**) from *Erythrina sigmoidea* Hua (Kuete et al., 2014c), isoneorautenol (**29**) from *Erythrina excelsa* Baker (Kuete et al., 2014d), xanthones: cudraxanthone I (**17**) from *Milicia excelsa* Welw C.C. Berg. (Kuete et al., 2013b) and xanthone V_1_ (**46**) from *Vismia laurentii* De Wild. (Kuete et al., 2011d). Activators of initiator caspases 8 and 9 include benzophenone **26** (Kuete et al., 2013d) and **30** (Kuete et al., 2013d), flavonid **18** (Kuete et al., 2015c), isoflavonoid **9** (Kuete et al., 2014c), or xanthone **17** (Kuete et al., 2013b).

**Table 1:** Cytotoxic plants of Central, East and West Africa and their molecular targets.

**Plant species and family/distribution in Central, East and West Africa**	**Traditional uses**	**Bioactive or potentially bioactive components**	Reported cytotoxic activity^*^	**Molecular targets and/or effects on resistant cells**
*Aframomum arundinaceum* (Oliver & Hanbury) K. Schum (Zinziberaceae)/Western and Central Africa	Anti-helmintic; against body odor; toothache; fungal infections (Tane et al., 2005)	Aframodial; 8(17),12-labdadien-15,16-dial; galanolactone; galanal A; galanal B; 1-*p*-menthene-3,6-diol; 1,4-dihydroxybenzene; naringenin; kaempferol-3,7,4′-trimethylether (Kuete et al., 2014a)	Cytotoxicity of fruit methanol extract toward CCRF-CEM cells, CEM/ADR5000 cells, MDA-MB-231-*pcDNA* cells, MDA-MB-231-*BCRP* cells, HCT116 (*p53^+/+^*) cells, HCT116 (*p53^−/−^*) cells, U87MG cells, U87MG.Δ*EGFR* cells, HepG2 cells (Kuete et al., 2014a)	Hypersensitivity: CEM/ADR5000 cells vs. CCRF-CEM cells (D.R. 0.76); Normal sensitivity: MDA-MB-231-*BCRP* cells vs. MDA-MB-231-*pcDNA* cells (D.R. 1.02); U87MG.Δ*EGFR* cells vs. U87MG cells (D.R. 0.95) (Kuete et al., 2014a)
*Aframomum polyanthum* K. Schum (Zinziberaceae)/Tropical Africa	Cancer (Kuete et al., 2014a, 2015b)	Aframodial (Ayafor et al., 1994)	Cytotoxicity of fruit methanol extract toward CCRF-CEM cells, CEM/ADR5000 cells, MDA-MB-231-*pcDNA* cells, MDA-MB-231-*BCRP* cells, U87MG.Δ*EGFR* cells (Kuete et al., 2014a, 2015b)	Hypersensitivity: MDA-MB-231-*BCRP* cells vs. MDA-MB-231-*pcDNA* cells (D.R. 0.89); U87MG.Δ*EGFR* cells vs. U87MG cells (D.R. < 0.51) (Kuete et al., 2014a)
*Albizia adianthifolia* (Schum.) (Fabaceae)/Angola (Angola), Benin, Cameroon, Central African Republic, Congo, DR Congo, Ivory Cost, Equatorial Guinea, Gabon, Gambia, Ghana, Guinea, Guinea-Bissau, Kenya, Liberia, Madagascar, Malawi, Mozambique, Nigeria, Rwanda, Senegal, Sierra Leone, Sudan, Tanzania, Togo, Uganda	Treatment of skin diseases, bronchitis, eyes inflammation, tapeworm, headaches and sinusitis (Watt and Breyer-Brandwyk, 1962; Van Wyk and Gericke, 2000	Adianthifoliosides A, B, D (Haddad et al., 2003, 2004), lupeol and aurantiamide acetate (Tamokou J. D. D. et al., 2012), prosapogenins (Haddad et al., 2002)	Cytotoxicity of the methanol extract from bark and roots toward CCRF-CEM cells, CEM/ADR5000 cells, MDA-MB-231-*pcDNA* cells, MDA-MB-231-*BCRP* cells, HCT116 (*p53^+/+^*) cells, HCT116 (*p53^−/−^*) cells, U87MG cells, U87MG.Δ*EGFR* cells, HepG2 cells (Kuete et al., 2016e)	Hypersensitivity: U87MG.Δ*EGFR* cells vs. U87MG cells (D.R.: 0.43 (bark extract) and 0.39 (roots extract)); Roots methanol extract induces apoptosis in CCRF-CEM cells through caspases activation and MMP loss (Kuete et al., 2016e)
*Alchornea cordifolia* (Schum. & Thonn.) Müll.-Arg. (Euphorbiaceae)/Tropical Africa from Senegal to Kenya and Tanzania and throughout Central Africa to Angola	Treat rheumatic pains, fever, wounds, diarrhea, convulsions, coughs, gonorrhea, yaws, ulcer, rheumatic pains, bronchial troubles (Ogungbamila and Samuelsson, 1990; Adeneye et al., 2014)	Alchornine, alchorneinone, gentisnic acid and yohimbine (Ogungbamila and Samuelsson, 1990)	Cytotoxicity of the methanol extract from bark and roots toward CCRF-CEM cells, CEM/ADR5000 cells, MDA-MB-231-*pcDNA* cells, MDA-MB-231-*BCRP* cells, HCT116 (*p53^+/+^*) cells, U87MG cells, U87MG.Δ*EGFR* cells (Kuete et al., 2016e)	Hypersensitivity: U87MG.Δ*EGFR* cells vs. U87MG cells (D.R.: 0.83 (leave extract) and < 0.40 (bark extract)); Leaves methanol extract induces apoptosis in CCRF-CEM cells through MMP loss and increase ROS production (Kuete et al., 2016e)
*Annona muricata* Lin. (Annonaceae)/Tropical Africa including Cameroon and Nigeria	Treatment of wounds and insomnia; antiparasitic, insecticidal (Rajeswari et al., 2012)	Epomuricenins-A and B, montecristin, cohibins-A and B, muridienins-1 and 2, muridienins-3 and 4, muricadienin and chatenaytrienins-1, 2 and 3 and sabadelin, murihexol, donhexocin, annonacin A and annonacin B (Rajeswari et al., 2012), Annomuricin E (Zorofchian Moghadamtousi et al., 2015)	Cytotoxicity of fruit pericarp, leave and seeds methanol extract toward CCRF-CEM cells and CEM/ADR5000 cells (Kuete et al., 2016b), HL60 cells, HL60AR cells, MDA-MB-231-*pcDNA* cells, MDA-MB-231-*BCRP* cells, HCT116 (*p53^+/+^*) cells, HCT116 (*p53^−/−^*) cells, U87MG cells, U87MG.Δ*EGFR* cells, HepG2 cells (Kuete et al., 2013c)	Induced apoptosis in CCRF-CEM cells mediated by MMP loss (Kuete et al., 2016b); Capsules consisted of 100% pure, finely milled leaf/stem powder of the plant with no binders or fillers induces necrosis of PC cells by inhibiting cellular metabolism, downregulated the expression of molecules related to hypoxia and glycolysis in PC cells (Torres et al., 2012); Ethyl acetate extract of leaves reduces the colonic aberrant crypt foci formation in rats and induced down-regulation of PCNA and Bcl-2 proteins and the up-regulation of Bax protein (Zorofchian Moghadamtousi et al., 2015)
*Anonidium mannii* (oliv) Engl. et Diels. (Anonaceae)/Central and West Africa, including the DR Congo, Congo, Central African Republic, Angola, Ghana, Nigeria, Gabon and Cameroon	Treatment of sore feet, spider bite, bronchitis, dysentery, sterility caused by poison, gastroenteritis (Thomas et al., 2003); syphilis, infectious diseases (Noumi and Eloumou, 2011); diarrhea, snake bite, malaria (Betti, 2004), cancer (Kuete et al., 2013a)	Alkaloids, phenols, saponins, tannins, sterols, triterpenes (Kuete et al., 2013a)	Cytotoxicity of the methanol extract from leaves toward CCRF-CEM cells, CEM/ADR5000 cells, MDA-MB-231-*pcDNA* cells, MDA-MB-231-*BCRP* cells, HCT116 (*p53^+/+^*) cells, HCT116 (*p53^−/−^*) cells, U87MG cells, U87MG.Δ*EGFR* cells, HepG2 cells (Kuete et al., 2013a)	Hypersensitivity: U87MG.Δ*EGFR* cells vs. U87MG cells (D.R.: < 0.41); Normal sensitivity: CEM/ADR5000 cells vs. CCRF-CEM cells (D.R.: 0.95); induces apoptosis in CCRF-CEM cells by disruption of MMP and increase ROS production (Kuete et al., 2013a)
*Anthocleista schweinfurthii* Gilg. (Loganiaceae)/Tropical Africa—Nigeria to Ethiopia, south to Angola, Zambia and Tanzania	Treatment of hernia, female sterility, stomach-ache in women, ovarian problems, venereal diseases, bronchitis, fever, purgative, malaria, hard abscesses anthelminthic, otitis, pain, malaria, cancers, venereal diseases, bacterial diseases (Ngbolua et al., 2014)	Polyphenols, alkaloids, terpenes and steroids (Ngbolua et al., 2014), schweinfurthiin 1, bauerenone 2, bauerenol 3, 1-hydroxy-3,7,8 trimethoxy-xanthone 4 and 1, 8-dihydroxy-3, 7 dimethoxy-xanthone 5 (Mbouangouere et al., 2007)	Cytotoxicity of fruit methanol extract toward CCRF-CEM cells, CEM/ADR5000 cells, MDA-MB-231-*pcDNA* cells, MDA-MB-231-*BCRP* cells, HCT116 (*p53^+/+^*) cells, HCT116 (*p53^−/−^*) cells (Kuete et al., 2016a)	Normal sensitivity: CEM/ADR5000 cells vs. CCRF-CEM cells (D.R. 1.11); HCT116 (*p53^−/−^*) vs. HCT116 (*p53^+/+^*) cells (D.R. 0.96) (Kuete et al., 2014a)
*Beilschmiedia acuta* Kosterm (Lauraceae)/Cameroon, Central African Republic	Treatment of cancer and gastrointestinal infections (Kuete et al., 2014e)	Flavonoids, triterpenes, phenols, saponins, alkaloids (Kuete et al., 2014e)	Cytotoxicity of the methanol extract from roots toward CCRF-CEM cells, CEM/ADR5000 cells, MDA-MB-231-*pcDNA* cells, MDA-MB-231-*BCRP* cells, HCT116 (*p53^+/+^*) cells, HCT116 (*p53^−/−^*) cells, U87MG cells, U87MG.Δ*EGFR* cells (Kuete et al., 2014e)	Hypersensitivity (leaves extract): HCT116 (*p53^−/−^*) cells vs. HCT116 (*p53^+/+^*) cells (D.R.: 0.23); induces apoptosis in CCRF-CEM cells (Kuete et al., 2014e)
*Calliandra portoricensis* (Jacq.) Benth. (Fabaceae)/Ghana, Nigeria, Uganda	Treatment of lumbago, pain relief, prostate diseases, and constipation, gonorrhea, headaches and ophthalmic preparation (Adaramoye et al., 2015)	Saponins, tannins, flavonoids and glycosides (Aguwa and Lawal, 1988)	Cytotoxicity of the root methanol extract toward PC-3 cells and LNCaP cells (Adaramoye et al., 2015)	Antiangiogenic activity *via* inhibition of of the growth of blood capillaries on the chicken chorioallantoic membrane, induces DNA fragmentation in PC-3 cells and LNCaP cells (Adaramoye et al., 2015)
*Dorstenia psilurus* Welwitch (Moraceae)/Tropical Africa including Angola, Cameroon, Uganda, Tanzania, Malawi, Mozambique	Treatment of arthralgia, cardiovascular disorders, rheumatism, snakebites, headache, stomach disorders, diuretic, tonic, stimulant, analgesic, cancer (Ruppelt et al., 1991; Adjanohoun et al., 1996; Ngadjui et al., 1998; Dimo et al., 2001; Kuete et al., 2011a)	Psoralen; 2-sitosterol glucoside analgesic (Ngadjui et al., 1998), dorsilurins C, F-K (Tabopda et al., 2008)	Cytotoxicity of twigs methanol extract toward CCRF-CEM cells and CEM/ADR5000 cells (Kuete et al., 2011a), HL-60 cells and PC-3 cells (Pieme et al., 2013)	Normal sensitivity: CEM/ADR5000 cells vs. CCRF-CEM cells (D.R. 0.88) (Kuete et al., 2011a), induces apoptosis on HL-60 cells by the generation of ROS, MMP loss, modification in the DNA distribution and enhance of G2/M phase cell cycle (Pieme et al., 2013)
*Echinops giganteus* var. lelyi (C. D. Adams) A. Rich. (Composiatae)/Cameroon, Ethiopia, Rwanda, Sudan, Tanzania, Uganda, DR Congo	Treatment of cancer, heart and gastric troubles (Tene et al., 2004; Kuete et al., 2011a)	Lupeol, sitosteryl, β-_D_-glucopyranoside oleanolide, tetrahydrofurano-ceramide, β-amyrin acetate (3), 2-(penta-1,3-diynyl)-5-(4-hydroxybut-1-ynyl)-thiophene, 2-(penta-1,3-diynyl)-5-(3,4-dihydroxybut-1-ynyl)-thiophene, 4-hydroxy-2,6-di-(3′,4′-dimethoxyphenyl)-3,7-dioxabicyclo-(3.3.0)octane (Tene et al., 2004; Sandjo et al., 2016), 2-(penta-1,3-diynyl)-5-(4-hydroxybut-1-ynyl)-thiophene, candidone, ursolic acid and 4-hydroxy-2,6-di-(3′,4′-dimethoxyphenyl)-3,7dioxabicyclo-(3.3.0)octane (Kuete et al., 2013c)	Cytotoxicity of rhizomes methanol extract toward CCRF-CEM cells and CEM/ADR5000 cells (Kuete et al., 2011a), HL60 cells, HL60AR cells, MDA-MB-231-*pcDNA* cells, MDA-MB-231-*BCRP* cells, HCT116 (*p53^+/+^*) cells, HCT116 (*p53^−/−^*) cells, U87MG cells, U87MG.Δ*EGFR* cells, HepG2 cells (Kuete et al., 2013c)	Hypersensitivity: HCT116 (*p53^−/−^*) cells vs. HCT116 (*p53^+/+^*) cells (D.R.: 0.82); Normal sensitivity: U87MG.Δ*EGFR* cells vs. U87MG cells (D.R. 0.92); (Kuete et al., 2013c); induces apoptosis in CCRF-CEM cells *via* the loss of MMP (Kuete et al., 2013c)
*Elaeophorbia drupifera* (Thonn.) Stapf. (Euphorbiaceae)/from Guinea east to Uganda and from DR Congo and Angola	Treatment of hypertension and diabetes (Eno and Azah, 2004)	Euphol, tirucallol, euphorbol, ingenol elaeophorbate, epitaraxerol, taraxerone, friedelin, lup-20(29)-en-3-one or lupenone, lupeol, olean-12-ene-3-one, olean-12-ene-3-ol, elaeophorbate (Kinghorn and Evans, 1974; Ahiahonu and Goodenowe, 2007), stigmasterol and β-sitosterol, sitosterol-*O*-β-_D_ -xylopyranoside, 3,3′,4′-tri-*O*-methylellagic acid, afzelin and quercetin-3-*O-β*-_D_-xylopyranoside, 3,3′,4′-tri-*O*-methylellagic acid 4-*O-β*-_D_-glucopyranoside, ellagic acid-4-*O-β*-xylopyranoside-3,3′,4′-trimethyl ether (Voukeng et al., 2017)	Cytotoxicity of the methanol extract from leaves toward CCRF-CEM cells, CEM/ADR5000 cells, MDA-MB-231-*pcDNA* cells, MDA-MB-231-*BCRP* cells, HCT116 (*p53^+/+^*) cells, HCT116 (*p53^−/−^*) cells, U87MG cells, U87MG.Δ*EGFR* cells, HepG2 cells (Kuete et al., 2013e)	Hypersensitivity: U87MG.Δ*EGFR* cells vs. U87MG cells (D.R.: 0.68); Normal sensitivity: CEM/ADR5000 cells vs. CCRF-CEM cells (D.R.: 1.12); HCT116 (*p53^−/−^*) cells vs. HCT116 (*p53^+/+^*) cells (D.R.: 1.13) (Kuete et al., 2013e)
*Enterolobium cyclocarpum* (Jacq.) Griseb.(Fabaceae)/West Africa	Treatment of inflammations, tumors, cold and bronchitis (Burkill, 1985)	_D_-Limonene, terpineol, eugenol and *d*-(+)-pinitol (Sowemimo et al., 2015)	Cytotoxicity of the methanol extract from leaves toward HeLa cells and MCF7 cells (Sowemimo et al., 2015)	Induces apoptosis and cells cycle arrest G2/M phase in HeLa cells and G1/G0 in MCF7 cells; causes phosphatidylserine translocation (Sowemimo et al., 2015)
*Erythrina sigmoidea* Hua (Fabaceae)/Cameroon, Chad	Used as antidotes (venomous stings, bites, etc.), diuretic, febrifuge and Treatment of arthritis, rheumatism, pulmonary troubles, stomach troubles, infectious diseases and kidney diseases (Burkill, 1985), gastrointestinal infections, venereal diseases and leprosy (Mabeku et al., 2011)	6α-hydroxyphaseollidin (**9**), atalantoflavone (**15**), bidwillon A (**16**), neobavaisoflavone (**35**), neocyclomorusin (**36**), and Sigmoidin I (**44**) (Kuete et al., 2014c) (Kuete et al., 2014c)	Cytotoxicity of bark methanol extract toward CCRF-CEM cells, CEM/ADR5000 cells, MDA-MB-231-*pcDNA* cells, MDA-MB-231-*BCRP* cells, HCT116 (*p53^+/+^*) cells, HCT116 (*p53^−/−^*) cells, U87MG cells, U87MG.Δ*EGFR* cells, HepG2 cells (Kuete et al., 2016a)	Hypersensitivity: HCT116 (*p53^−/−^*) cells vs. HCT116 (*p53^+/+^*) cells (D.R. 0.83); U87MG.Δ*EGFR* cells vs. U87MG cells (D.R. 0.66); Normal sensitivity: CEM/ADR5000 cells vs. CCRF-CEM cells (D.R. 1.08); induces apoptosis in CCRF-CEM leukemia cells *via* disruption of the MMP (Kuete et al., 2014a)
*Gladiolus quartinianus* A. Rich (Iridaceae)/Cameroon, Senegal to Ethiopia	Treatment of gastrointestinal infections and cancer (Kuete et al., 2013a)	Alkaloids, anthocyanins, anthraquinones, phenols, saponins, tannins, sterols, triterpenes (Kuete et al., 2013a)	Cytotoxicity of the methanol extract from whole plant toward CCRF-CEM cells, CEM/ADR5000 cells, MDA-MB-231-*pcDNA* cells, MDA-MB-231-*BCRP* cells, HCT116 (*p53^+/+^*) cells, HCT116 (*p53^−/−^*) cells, U87MG.Δ*EGFR* cells (Kuete et al., 2013a)	Hypersensitivity: U87MG.Δ*EGFR* cells vs. U87MG cells (D.R.: < 0.85); Normal sensitivity: HCT116 (*p53^−/−^*) cells vs. HCT116 (*p53^+/+^*) cells (D.R.: 1.12); induces apoptosis in CCRF-CEM cells by disruption of MMP (Kuete et al., 2013a)
*Imperata cylindrica* Beauv. var. koenigii Durand et Schinz (Poaceae)/Benin, Burkina Faso, DR Congo, Ivory Cost, Gambia, Ghana, Guinea, Kenya, Liberia, Mali, Mozambique, Niger, Nigeria, Senegal, Tanzania, Togo, Uganda	Used as diuretic and anti-inflammatory and cancer agent (Nishimoto et al., 1968; Kuete et al., 2011a)	Jaceidin, quercetagetin-3, 5, 6, 3.′-tetramethyl ether, β-Sitosterol-3-0-β-_D_-glucopyranosy1-6″- tetradecanoate (Mohamed et al., 2009), imperanene (Matsunaga et al., 1995)	Cytotoxicity of roots methanol extract toward CCRF-CEM cells and CEM/ADR5000 cells, MiaPaca-2(Kuete et al., 2011a), HL60 cells, HL60AR cells, MDA-MB-231-*pcDNA* cells, MDA-MB-231-*BCRP* cells, HCT116 (*p53^+/+^*) cells, HCT116 (*p53^−/−^*) cells, U87MG cells, U87MG.Δ*EGFR* cells, HepG2 cells (Kuete et al., 2013c); cytotoxicity of leaves methanol extract against SCC-9 cells (Keshava et al., 2016) and against HT-29 cells (Kwok et al., 2016)	Hypersensitivity: CEM/ADR5000 cells vs. CCRF-CEM (D.R. 0.90) cells (Kuete et al., 2011a), apoptosis in CCRF-CEM cells *via* the loss of MMP (Kuete et al., 2013c); leaves methanol extract reduced the clonogenic potential and inhibited cell proliferation by arresting the cell cycle in the G2/M phase in SCC-9 cells as well as DNA fragmentation (Keshava et al., 2016); Induced G2/M arrest and apoptosis in HT-29 cells mediated by caspase 3/7 activation and ROS production (Kwok et al., 2016)
*Markhamia tomentosa* (Benth.) K.Schumex.Engl.(Bignoniaceae)/West Africa	Treatment of oedema, cancer, gout and scrotal elephantiasis, pulmonary troubles and general body pain (Burkill, 1985; Ibrahim et al., 2013)	Pomolic acid, oleanolic acid, tormentic acid, and β-sitosterol, paulownin, palmitone, palustrine, 2-acetylnaphtho[2,3-b] furan-4,9-dione, 2-acetyl-6- methoxy-naphtho[2,3-b] furan-4,9-dione, luteolin, luteolin-7-rutinoside, and luteolin-3′,7-di-*O*-glucoside (Ibrahim et al., 2016)	Cytotoxicity of the methanol extract from leaves toward HeLa cells (Ibrahim et al., 2013)	Induces apoptosis and cell cycle arrest in HeLa cells in the G0/G1; induces phosphatidylserine translocation and depolarization MMP (Ibrahim et al., 2013)
*Morus mesozygia* Stapf. (Moraceae)/Tropical Africa, from Senegal eastward to Ethiopia and southward to Zambia, Angola, Mozambique	Treatment of arthritis, rheumatism, malnutrition, debility, pain-killers, stomach disorders, wound infections, gastroenteritis, peptic ulcer, infectious diseases (Burkill, 1985; Kuete and Efferth, 2010, 2011)	moracins Q-U, 3beta-acetoxyurs-12-en-11-one, marsformoxide, moracin C, moracin M, moracin K, artocarpesin, cycloartocarpesin, morachalcone A (Kapche et al., 2009; Kuete et al., 2009); kushenol E, artochamin C, moracin C and moracin L (Nicolle et al., 2009)	Cytotoxicity of bark methanol extract toward CCRF-CEM cells, CEM/ADR5000 cells, MDA-MB-231-*pcDNA* cells, MDA-MB-231-*BCRP* cells, HCT116 (*p53^+/+^*) cells, HCT116 (*p53^−/−^*) cells, U87MG cells, U87MG.Δ*EGFR* cells, HepG2 cells (Kuete et al., 2016a)	Normal sensitivity: CEM/ADR5000 cells vs. CCRF-CEM cells (D.R. 1.04); HCT116 (*p53^−/−^*) cells vs. HCT116 (*p53^+/+^*) cells (D.R. 0.95); U87MG.Δ*EGFR* cells vs. U87MG cells (D.R. 1.06) (Kuete et al., 2014a)
*Nauclea latifolia* Smith. (Rubiaceae)/West tropical Africa—from Ghana to Gabon and DR Congo	Treatment of gonorrhea (Abbiw, 1990), hypertension (Akabue and Mittal, 1982), gastrointestinal tract disorders (Madubunyi, 1995), prolong menstrual flow (Elujoba, 1995), stomach pain, constipation, fever, diarrhea, dysentery (Anowi et al., 2012)	Naucleamides A,B,C,D,E (Shigemori et al., 2003)	Cytotoxicity of bark and leave methanol extract toward CCRF-CEM cells, CEM/ADR5000 cells, MDA-MB-231-*pcDNA*, cells, MDA-MB-231-*BCRP* cells, HCT116 (*p53^+/+^*) cells, HCT116 (*p53^−/−^*) cells (Kuete et al., 2016a)	Hypersensitivity: MDA-MB-231-*BCRP* cells vs. MDA-MB-231-*pcDNA* cells (D.R. 0.80); HCT116 (*p53^−/−^*) cells vs. HCT116 (*p53^+/+^*) cells (D.R. 0.88); Normal sensitivity: CEM/ADR5000 cells vs. CCRF-CEM cells (D.R. 0.98) (Kuete et al., 2014a)
*Nauclea pobeguinii* (Pobég. ex Pellegr.) Merr. ex E.M.A. (Rubiaceae)/South Tropical Africa: Angola, Zambia, West Tropical Africa: Burkina, Ghana, Guinea, Guinea-Bissau, Ivory Coast, Nigeria, Senegal, Sierra Leone, West-Central Tropical Africa: Cameroon Central African Republic, Congo, DR Congo, Gabon	Used as abortive, Treatment of stomach ache, infectious diseases (Karou et al., 2011), jaundice (Kadiri et al., 2007), fever, diarrhea, worm, malaria (Mesia et al., 2005)	Nauclefine 1 and 2, strictosamide, carboxystrictosidine, methylangustoline, 3-*O*-β-*D*-fucosyl-quinovic-acid, 3-keto-quinovic-acid (Karou et al., 2011); angustoline (Zeches et al., 1985), 3-acetoxy-11-oxo-urs-12-ene, *p*-coumaric acid, citric acid trimethyl ester, resveratrol, resveratrol *β-*_D_*-*glucopyranoside, strictosamide (Kuete et al., 2015f)	Cytotoxicity of the methanol extract from bark and leaves toward CCRF-CEM cells, CEM/ADR5000 cells, MDA-MB-231-*pcDNA* cells, MDA-MB-231-*BCRP* cells, HCT116 (*p53^+/+^*) cells, HCT116 (*p53^−/−^*) cells, U87MG cells, U87MG.Δ*EGFR* cells (Kuete et al., 2015f)	Hypersensitivity (bark extract): CEM/ADR5000 cells vs. CCRF-CEM cells (D.R.: 0.80); MDA-MB-231-*BCRP* cells vs. MDA-MB-231-*pcDNA* cells (D.R.: 0.53); HCT116 (*p53^−/−^*) cells vs. HCT116 (*p53^+/+^*) cells (D.R.: 0.54); U87MG.Δ*EGFR* cells vs. U87MG cells (D.R.: 0.47)
*Pachypodanthium staudtii* Engl & Diels (Annonaceae)/Sierra Leone east to the Central African Republic and south to Gabon and DR Congo	Treatment of cancer, chest pain (Irvine, 1961); bronchitis (Bouquet and Debray, 1974) and oedema (Ngadjui et al., 1989)	Pachypodol, 2,4,5-Trimethoxystyrene, Pachypophyllin, pachypostaudins A and B (Ngadjui et al., 1989); Sabinene, β -elemene, *E*- β -caryophyllene, β -selinene, β -bisabolene, δ -cadinene, 2,4,5-trimethoxy-1-vinylbenzene (Yapi et al., 2012)	Cytotoxicity of leave, bark and roots methanol extract toward CCRF-CEM cells, CEM/ADR5000 cells, MDA-MB-231-*pcDNA* cells, MDA-MB-231-*BCRP* cells, HCT116 (*p53^+/+^*) cells, HCT116 (*p53^−/−^*) cells, U87MG cells, U87MG.Δ*EGFR* cells, HepG2 cells (Kuete et al., 2016b)	Hypersensitivity: CEM/ADR5000 cells vs. CCRF-CEM cells (D.R. 0.87); MDA-MB-231-*BCRP* cells vs. MDA-MB-231-*pcDNA* cells (D.R. 0.90); Normal sensitivity: U87MG.Δ*EGFR* cells vs. U87MG cells (D.R. 1.05) (Kuete et al., 2016b)
*Passiflora edulis* Sims (Passifloraceae)/Central and East including Cameroon, Tanzania, Uganda	Treatment of cancer, fungal infections, inflammation, insomnia and anxiety, antihypertensive (Ichimura et al., 2006), gastric trouble (Silva et al., 2006), antioxidant (Kannan et al., 2011)	Ionone-I, ionone-II, megastigma-5,8-dien-4-1, megastigma-5,8(*Z*)-diene-4-1, 4,4*a*-Epoxy-4, 4*a*-dihydroedulan, 3-hydroxyedulan, edulan-I, edulan-II, passifloric acid methyl ester (Kannan et al., 2011)	Cytotoxicity of fruit pericarp and fruit methanol extract toward CCRF-CEM cells and CEM/ADR5000 cells (Kuete et al., 2016b)	Induces apoptosis in CCRF-CEM cells mediated by MMP loss (Kuete et al., 2016b); fruit juice reduces the number, size, and invasiveness of transformed foci in a BALB/c 3T3 neoplastic transformation model; activated caspase-3 in MOLT-4 cells (Rowe et al., 2004)
*Piper capense* L.f. (Piperaceae)/from Guinea to Ethiopia and south to Angola, Mozambique	Sleep inducing remedy, anthelmintic, anticancer (Kokowaro, 1976; Van Wyk and Gericke, 2000; Kuete et al., 2011a)	Kaousine, *Z*-antiepilepsirine (Kaou et al., 2010), piperine, 4,5-dihydropiperine (Pedersen et al., 2009), beta-pinene, sabinene, alpha-pinene (Woguem et al., 2013)	Cytotoxicity of seeds methanol extract toward CCRF-CEM cells and CEM/ADR5000 (Kuete et al., 2011a), MDA-MB 231 cells, A375 cells, HCT116 cells (Woguem et al., 2013), HL60 cells, HL60AR cells, MDA-MB-231-*pcDNA* cells, MDA-MB-231-*BCRP* cells, HCT116 (*p53^+/+^*) cells, HCT116 (*p53^−/−^*) cells, U87MG cells, U87MG.Δ*EGFR* cells, HepG2 cells (Kuete et al., 2013c)	Hypersensitivity: CEM/ADR5000 cells vs. CCRF-CEM cells (D.R. 0.90) (Kuete et al., 2011a), apoptosis in CCRF-CEM cells *via* the loss of MMP and increase ROS production (Kuete et al., 2013c)
*Polyscias fulva* (Hiern) Harms. (Araliaceae)/Tropical Africa - Sierra Leone to Sudan, Ethiopia to Angola, Zambia and Mozambique	Malaria, fever, mental illness (Tshibangu et al., 2002); venereal infections and obesity (Jeruto et al., 2007; Focho et al., 2009) and cancer (Kuete et al., 2014e)	Polysciasoside A, kalopanax-saponin B, alpha-hederin (Bedir et al., 2001; Kuete and Efferth, 2011)	Cytotoxicity of the methanol extract from roots and leaves toward CCRF-CEM cells, CEM/ADR5000 cells, MDA-MB-231-*pcDNA* cells, MDA-MB-231-*BCRP* cells, HCT116 (*p53^+/+^*) cells, HCT116 (*p53^−/−^*) cells, U87MG cells, U87MG.Δ*EGFR* cells, HepG2 cells (Kuete et al., 2014e)	Hypersensitivity: HCT116 (*p53^−/−^*) cells vs. HCT116 (*p53^+/+^*) cells (D.R.: 0.41); induces apoptosis in CCRF-CEM cells via the alteration of MMP and enhanced ROS production (Kuete et al., 2014e)
*Sclerocarya birrea* (A. Rich.) Hochst. (Anacardiaceae)/throughout most of sub-Saharan Africa outside the humid forest zone, from Mauritania and Senegal to Ethiopia and Eritrea, Namibia, Botswana, Mozambique	Treatment of stomach aches, diarrhea, wounds, coughs (Gouwakinnou et al., 2011)	Quercetin 3-*O*-alpha-l-(5″-galloyl)-arabinofuranoside, quercetin 3-*O*- β-_D_-(6″-galloyl)glucopyranoside, quercetin 3- *O*- β-_D_–(6″- galloyl)galactopyranoside, quercetin 3-*O*-α-_L_-rhamnopyranoside, kaempferol 3- *O*- β-_D_-(6″-galloyl)glucopyranoside, quercetin 3- β-_D_–glucopyranoside, myricetin 3- *O*- α-_L_-rhamnopyranoside, and kaempferol 3- *O*- α-_L_-rhamnopyranoside, gallic acid, (-)-epicatechin 3-*O*-galloyl ester, (-)-epigallocatechin 3-*O*-galloyl ester (Braca et al., 2003), terpinen-4-ol, pyrrolidine, aromadendrene, α-gurjunene (Njume et al., 2011)	Cytotoxicity of the methanol extract from roots toward HepG2 cells (Armentano et al., 2015)	Induces apoptosis *via* ROS production in HepG2 cells (Armentano et al., 2015)
*Tridesmostemon omphalocarpoides* Engl. (Sapotaceae)/Cameroon, Gabon, Congo, DR Congo	Treatment of gastroenteritis and skin lesions (Kuete et al., 2006)	Alkaloids, phenols, polyphenols, saponins, tannins, triterpenes, anthraquinones and steroids (Kuete et al., 2006)	Cytotoxicity of bark methanol extract toward CCRF-CEM cells, CEM/ADR5000 cells, MDA-MB-231-*pcDNA* cells, HCT116 (*p53^+/+^*) cells, HCT116 (*p53^−/−^*) cells, U87MG cells (Kuete et al., 2016a)	Normal sensitivity: CEM/ADR5000 cells vs. CCRF-CEM cells (D.R. 0.99); HCT116 (*p53^−/−^*) cells vs. HCT116 (*p53^+/+^*) cells (D.R. 1.15) (Kuete et al., 2014a)
*Uapaca togoensis* Pax (Euphorbiaceae)/Tropical Africa from Sierra Leone to DR Congo; Predominant in Cameroon	Antiemetic, lotion for skin disorders (Mengome et al., 2010), remedy for pneumonia, cough, fever, rheumatism, vomiting, epilepsy (Kone et al., 2006) and bacterial diseases (Kone et al., 2004)	β-amyryl acetate, 11-oxo-α-amyryl acetate, lupeol, pomolic acid, futokadsurin B, arborinin, 3-*O-β-*_D_-glucopyranosyl sitosterol (Kuete et al., 2015e)	Cytotoxicity of the methanol extract from fruit toward CCRF-CEM cells, CEM/ADR5000 cells, MDA-MB-231-*pcDNA* cells, MDA-MB-231-*BCRP* cells, HCT116 (*p53^+/+^*) cells, HCT116 (*p53^−/−^*) cells, U87MG cells, U87MG.Δ*EGFR* cells, HepG2 cells (Kuete et al., 2015e)	Hypersensitivity: MDA-MB-231-*BCRP* cells vs. MDA-MB-231-*pcDNA* cells (D.R.: 0.16); HCT116 (*p53^−/−^*) cells vs. HCT116 (*p53^+/+^*) cells (D.R.: 0.84); Normal sensitivity: CEM/ADR5000 cells vs. CCRF-CEM cells (D.R.: 1.05); U87MG.Δ*EGFR* cells vs. U87MG cells (D.R.: 1.08); induces apoptosis in CCRF-CEM cells by MMP loss (Kuete et al., 2015e)
*Vepris soyauxii* Engl. (Rutaceae)/Throughout West Africa, from Sierra Leone, Liberia, Ivory Cost, Mali, Ghana to Nigeria and Cameroon	Anti-fibriomyoma, Treatment of stomachache, malaria (Momeni et al., 2010) and cancer (Kuete et al., 2013a)	Alkaloids, anthocyanins, phenols, tannins, sterols, triterpenes (Kuete et al., 2013a)	Cytotoxicity of the methanol extract from leaves toward CCRF-CEM cells, CEM/ADR5000 cells, MDA-MB-231-*pcDNA* cells, MDA-MB-231-*BCRP* cells, HCT116 (*p53^+/+^*) cells, HCT116 (*p53^−/−^*) cells, U87MG cells, U87MG.Δ*EGFR* cells, HepG2 cells (Kuete et al., 2013a)	Hypersensitivity: U87MG.Δ*EGFR* cells vs. U87MG cells (D.R.: 0.47); Normal sensitivity: HCT116 (*p53^−/−^*) cells vs. HCT116 (*p53^+/+^*) cells (D.R.: 1.12); induces apoptosis in CCRF-CEM cells mediated by disruption of MMP (Kuete et al., 2013a)
*Xylopia aethiopica* (Dunal) A. Rich.(Annonaceae)/Angola, Benin, Burkina Faso, Cameroon, Central African Republic, DR Congo, Ethiopia, Gabon, Gambia, Ghana, Guinea, Guinea-Bissau, Ivory Coast, Kenya, Liberia, Mozambique, Nigeria, São Tomé and Príncipe, Senegal, Sierra Leone, Sudan, South Sudan, Tanzania, Togo, Uganda	Treatment of cancer, constipation; uterine hemorrhage, diuretic, fever (Iwu, 1993; Kuete et al., 2011a; Okafor, 2012)	Volatile oil (Tatsadjieu et al., 2003), xylopic acid (Osafo and Obiri, 2016), 6α-hydroxy-ent-kauran-19-oic acid, 3,4′,5-trihydroxy-6″,6″-dimethylpyrano[2,3-g]flavone, isotetrandrine (**51**) and trans-tiliroside (Kuete et al., 2015g),	Cytotoxicity of seeds methanol extract toward CCRF-CEM cells and CEM/ADR5000 cells (Kuete et al., 2011a), C-33A cells, KB cells, MCF-7 cells (Adaramoye et al., 2011), HL60 cells, HL60AR cells, MDA-MB-231-*pcDNA* cells, MDA-MB-231-*BCRP* cells, HCT116 (*p53^+/+^*) cells, HCT116 (*p53^−/−^*) cells, U87MG cells, U87MG.Δ*EGFR* cells, HepG2 cells (Kuete et al., 2013c)	Hypersensitivity: U87MG.Δ*EGFR* vs. U87MG (D.R. 0.53); Normal sensitivity: HCT116 (*p53^−/−^*) cells vs. HCT116 (*p53^+/+^*) cells (D.R.: 1.05)(Kuete et al., 2013c)(Kuete et al., 2013c)(Kuete et al., 2013c)(Kuete et al., 2013c); induces apoptosis in C-33A cells, nuclear fragmentation, cells accumulation in sub-G0/G1, cycle arrest in G2, up-regulation of p53 and p21 genes, and an increase in the Bax/Bcl-2 ratio (Adaramoye et al., 2011), apoptosis in CCRF-CEM cells *via* the loss of MMP (Kuete et al., 2013c)
*Zanthoxylum usambarense* (Engl.) Kokwaro (Rutaceae)/East tropical Africa - Ethiopia, Kenya, Tanzania, eastern DR Congo	Treatment of malaria, upper respiratory tract infections, cough, rheumatism, tooth decay (Ozkan et al., 2013)	Canthin-6-one, pellitorine, oxychelerythrine, norchelerythrine, (+)-sesamin, (+)-piperitol-3,3-dimethylallyl ether (He et al., 2002)	Cytotoxicity of the aqueous-methanol 70% extract from aeral part toward MDA-MB-231 cells and MCF-7 cells (Ozkan et al., 2013)	Induces apoptosis in MCF7 cells (Ozkan et al., 2013)
*Zinziber officinale Roscoe* (Zingiberaceae)/Tropical Africa	Treatment of infectious diseases, respiratory tract infections, cancer, indidigestion, diarrhea, nausea (Akoachere et al., 2002; Kato et al., 2006; Sakpakdeejaroen and Itharat, 2009; Kuete et al., 2011a)	2-(4-hydroxy-3-methoxyphenyl)ethanol and 2-(4-hydroxy-3-methoxyphenyl)ethanoic acid (Kato et al., 2006), 6-shogaol (Kim et al., 2008), zingiberene, camphene, β-sesquiphellandrene, β-bisabolene, α-farmesene, curcumene, cineole, citral, terpineol, terpenes, borneol, β-elemene, zingiberenol, limonene, geraniol, zingiberol, linalool (Chrubasik et al., 2005; Ali et al., 2008; Mbaveng and Kuete, 2017)	Cytotoxicity of rhizomes methanol extract toward CCRF-CEM cells and CEM/ADR5000 cells, MiaPaca-2 cells (Kuete et al., 2011a), CL-6 cells (Plengsuriyakarn et al., 2012); cytotoxicity of essential oil against HeLa cells (Santos et al., 2016)	Hypersensitivity: CEM/ADR5000 cells vs. CCRF-CEM cells (D.R. 0.88) (Kuete et al., 2011a); ethanol extract induces DNA fragmentation and up-regulation of MDR1 and MRP3 genes in CL-6 cells (Plengsuriyakarn et al., 2012)

* *Reported cell lines: leukemia cells [CCRF-CEM, CEM/ADR5000, HL60, and HL60AR]; Carcinoma cells [A375 melanoma cells; C-33A and Caski cervix carcinoma cells; CL-6 cholangiocarcinoma cells; MDA-MB-231-pcDNA3 and MDA-MB-231-BCRP clone 23 breast cancer cells; HT-29, HCT116 (p53^+/+^) and HCT116 (p53^−/−^) colon cancer cells; KB and SCC-9 human oral squamous carcinoma cells; U87MG and U87MG.ΔEGFR glioblastoma cells; HeLa cervical carcinoma; HepG2 hepatocarcinoma; PC-3, MiaPaca-2 pancreatic cancer cells; LNCaP human prostatic adenocarcinoma, AML12 normal hepatocytes; BALB/c 3T3 fibroblasts]; D.R.: degree of resistance; D.R. is determined as the ratio of IC_50_ value in the resistant divided by the IC_50_ in the sensitive cell line; AML12, HL60AR, CEM/ADR5000, MDA-MB-231-BCRP, HCT116 (p53^−/−^) and U87MG.ΔEGFR were used as the corresponding resistant counterpart for HepG2, HL60, CCRF-CEM, MDA-MB-231-pcDNA, HCT116 (p53^+/+^), U87MG, respectively; Hypersensitivity, D.R. < 0.90; Normal sensitivity, D.R. 1 to 1.19; MMP, mitochondrial membrane potential; ROS, reactive oxygen species; (-), not reported*.

**Table 2:** Bioactive compounds identified in cytotoxic plants of Central, East and West Africa and their molecular targets.

**Classes and compounds**	**Plant sources**	**Reported cytotoxic activity^a^**	**Molecular targets and/or effects on resistant cells**
**TERPENOIDS**			
Alpha-hederin (triterpene glycoside; **1**)	*Polyscias fulva* (Hiern) Harms. (Araliaceae) (Kuete et al., 2014e); *Clematis ganpiniana* L. (Ranunculaceae), *Hedera* spp. *Nigella* spp. (Cheng et al., 2014)	Cytotoxicity toward CCRF-CEM cells, CEM/ADR5000 cells, MDA-MB-231-*pcDNA* cells, MDA-MB-231-*BCRP* cells, HCT116 (*p53^+/+^*) cells, HCT116 (*p53^−/−^*) cells, U87MG cells, U87MG.Δ*EGFR* cells, HepG2 cells (Kuete et al., 2014e)	Genes closely associated with the response to alpha-hederin belong to diverse functional groups such as apoptosis, growth and cell cycle regulation, signal transduction, transcription, transport processes, nerve cell functions (Kuete et al., 2014e); induces disruption of MMP, caspase-3 activation, and increases the production of ROS in P388 cells, caspase-3 activation (Swamy and Huat, 2003); induces apoptosis in breast cancer cells, induces depolarization of MMP, promotes caspase-3 and caspase-9 activation. (Cheng et al., 2014); induces apoptosis, membrane permeabilization and morphologic changes in cancer cell lines through a cholesterol-dependent mechanism (Lorent et al., 2016); other molecular targets as identified by molecular docking simulations include Ras related protein rap-2a, cathepsin K, estradiol 17-beta dehydrogenase-1, GTPase HRas, cellular retinoic acid-binding protein 2, dihydroorate dehydrogenase (Sridhar et al., 2014)
Galanal A (diterpene; **2**)	*Aframomum arundinaceum (Oliver & Hanbury) K. Schum* (Zinziberaceae)(Kuete et al., 2014a)	Cytotoxicity toward CCRF-CEM cells, MDA-MB-231-*BCRP* cells (Kuete et al., 2014a)	Hypersensitivity: MDA-MB-231-*BCRP* cells vs. MDA-MB-231-*pcDNA* cells (D.R. < 0.70) (Kuete et al., 2014a); induces apoptosis in Jurkat human T-cell leukemia cells through DNA fragmentation, MMP alteration and caspase-3 activation as well as downregulation of the anti-apoptotic Bcl-2 protein (Miyoshi et al., 2003)
**PHENOLICS**			
2,2′,5,6′-tetrahydroxybenzophenone (benzophenone; **3**)	*Hypericum lanceolatum* Lam. (Hypericaceae)(Kuete et al., 2013d)	Cytotoxicity toward CCRF-CEM cells, CEM/ADR5000 cells, MDA-MB-231-*pcDNA* cells, MDA-MB-231-*BCRP* cells, HCT116 (*p53^+/+^*) cells, HCT116 (*p53^−/−^*) cells, U87MG cells, U87MG.Δ*EGFR* cells, HepG2 cells (Kuete et al., 2013d)	Hypersensitivity: U87MG.Δ*EGFR* cells vs. U87MG cells (D.R. 0.24) (Kuete et al., 2013d)
2-acetylfuro-1,4-naphthoquinone (naphthoquinone; **4**)	*Newbouldia laevis* Seems. (Bignoniaceae) (Eyong et al., 2006; Kuete et al., 2007a)	Cytotoxicity toward CCRF-CEM cells, CEM/ADR5000 cells, PF-382 cells, HL-60 cells, MiaPaCa-2 cells, Capan-1 cells, MCF-7 cells, SW-680 cells, 786-0 cells, U87MG cells, A549 cells, Colo-38 cells, HeLa cells, Caski cells (Kuete et al., 2011d)	Anti-angiogenic effects through inhibition of the growth of blood capillaries on the chorioallantoic membrane of quail eggs; induces apoptosis and cell cycle arrest in S-phase in CCRF-CEM (Kuete et al., 2011d)
3,4′,5-trihydroxy-6″,6″-dimethylpyrano[2,3-g]flavone (flavonoid; **5**)	*Xylopia aethiopica* (Dunal) A.Rich. (Annonaceae) (Kuete et al., 2015g)	Cytotoxicity toward CCRF-CEM cells, MDA-MB-231-*pcDNA* cells, MDA-MB-231-*BCRP* cells, HCT116 (*p53^+/+^*) cells, HCT116 (*p53^−/−^*) cells, U87MG cells, U87MG.Δ*EGFR* cells, HepG2 cells (Kuete et al., 2015g)	Normal sensitivity: HCT116 (*p53^−/−^*) cells vs. HCT116 (*p53^+/+^*) cells (D.R. 0.96); U87MG.Δ*EGFR* cells vs. U87MG cells (D.R. 1.03); induces apoptosis in CCRF-CEM cells, mediated by MMP disruption (Kuete et al., 2015g)
4′-hydroxy-2′,6′-dimethoxychalcone (flavonoid; **6**)	*Polygonum limbatum* Meisn. (Polygonaceae) (Kuete et al., 2014b)	Cytotoxicity toward CCRF-CEM cells, CEM/ADR5000 cells, MDA-MB-231-*pcDNA* cells, MDA-MB-231-*BCRP* cells, HCT116 (*p53^+/+^*) cells, HCT116 (*p53^−/−^*) cells, U87MG cells, U87MG.Δ*EGFR* cells, HepG2 cells (Kuete et al., 2014b)	Hypersensitivity: CEM/ADR5000 cells vs. CCRF-CEM cells (D.R.0.27); MDA-MB-231-*BCRP* cells vs. MDA-MB-231-*pcDNA* cells (D.R.0.33); Normal sensitivity: -231-*BCRP* cells vs. MDA-MB-231-*pcDNA* cells (D.R. 0.92); U87MG.Δ*EGFR* cells vs. U87MG cells (D.R. 1.17); Induced cell cycle arrest between Go/G1 phase and apoptosis in CCRF-CEM cells *via* disruption of MMP and increase ROS production (Kuete et al., 2014b)
4-hydroxylonchocarpin (flavonoid; **7**)	*Dorstenia barteri* (Mbaveng et al., 2008; Kuete et al., 2010)	Cytotoxicity toward CCRF-CEM cells, CEM/ADR5000 cells, HL-60 cells, MiaPaCa-2 cells, MCF-7 cells, 786-0 cells, U87MG cells, Colo-38 cells, HeLa cells, Caski cells (Kuete et al., 2011b)	Induces apoptosis in CCRF-CEM cells *via* caspase 3/7 activation; anti-angiogenic effect *via* inhibition of the growth of blood capillaries on the chorioallantoic membrane of quail eggs (Kuete et al., 2011b)
6,8-diprenyleriodictyol (flavonoid; **8**)	*Dorstenia mannii* Hook.f. (Ngadjui et al., 2000; Mbaveng et al., 2012)	Cytotoxicity toward CCRF-CEM cells, CEM/ADR5000 cells, PF-382 cells, HL-60 cells, MiaPaCa-2 cells, MCF-7 cells, 786-0 cells, U87MG cells, A549 cells, Colo-38 cells, HeLa cells, Caski cells (Kuete et al., 2011b)	Induces apoptosis in CCRF-CEM cells *via* caspase 3/7 activation; anti-angiogenic effect *via* inhibition of the growth of blood capillaries on the chorioallantoic membrane of quail eggs (Kuete et al., 2011b)
8-hydroxycudraxanthone G (xanthone; **10**)	*Garcinia nobilis* Engl. (Guttiferae) (Fouotsa et al., 2012, 2013)	Cytotoxicity toward CCRF-CEM cells, MDA-MB-231-*pcDNA* cells, MDA-MB-231-*BCRP* cells, HCT116 (*p53^+/+^*) cells, HCT116 (*p53^−/−^*) cells, U87MG cells, U87MG.Δ*EGFR* cells, HepG2 cells (Kuete et al., 2013b)	Hypersensitivity: MDA-MB-231-*BCRP* cells vs. MDA-MB-231-*pcDNA* cells (D.R. 0.74); HCT116 (*p53^−/−^*) cells vs. HCT116 (*p53^+/+^*) cells (D.R. 0.90); U87MG.Δ*EGFR* cells vs. U87MG cells (D.R. 0.55) (Kuete et al., 2013b)
Alpinumisoflavone (flavonoid; **12**)	*Ficus chlamydocarpa* Mildbr. & Burret (Moraceae) (Kuete et al., 2008)	Cytotoxicity toward CCRF-CEM cells, CEM/ADR5000 cells, MDA-MB-231-*pcDNA* cells, MDA-MB-231-*BCRP* cells, HCT116 (*p53^+/+^*) cells, HCT116 (*p53^−/−^*) cells, U87MG cells, U87MG.Δ*EGFR* cells, HepG2 cells (Kuete et al., 2016c)	Hypersensitivity: CEM/ADR5000 cells vs. CCRF-CEM cells (D.R.0.62); HCT116 (*p53^−/−^*) cells vs. HCT116 (*p53^+/+^*) cells (D.R. 0.86); U87MG.Δ*EGFR* cells vs. U87MG cells (D.R. 0.90); induces apoptosis in CCRF-CEM cells, mediated by loss of MMP and increase ROS production (Kuete et al., 2016c); induces apoptotic cell death in H2108 and H1299 cells, mediated by caspase 3/7 activation (Namkoong et al., 2011); induces apoptosis in esophageal squamous cell carcinoma by modulating miR-370/PIM1 signaling (Han et al., 2016); increases the expression of microRNA precursor, miR-101 by suppressing Protein Kinase B (Akt) signaling in renal cell carcinoma (Wang et al., 2017)
Amentoflavone (flavonoid; **13**)	*Dorstenia barteri* Bureau (Moraceae) (Mbaveng et al., 2008; Kuete et al., 2010)	Cytotoxicity toward CCRF-CEM cells, CEM/ADR5000 cells, MDA-MB-231-*pcDNA* cells, MDA-MB-231-*BCRP* cells, HCT116 (*p53^+/+^*) cells (Kuete et al., 2016c); MCF-7 cells (Chen et al., 2015), B16F-10 cells (Guruvayoorappan and Kuttan, 2008) and SW480 cells (Yang et al., 2014)	Hypersensitivity: CEM/ADR5000 cells vs. CCRF-CEM cells (D.R.0.77); MDA-MB-231-*BCRP* cells vs. MDA-MB-231-*pcDNA* cells (D.R.0.49); Normal sensitivity: HCT116 (*p53^−/−^*) cells vs. HCT116 (*p53^+/+^*) cells (D.R. 1.08) (Kuete et al., 2016c); downregulates cytokines mediated cyclooxygenase-2 and inducible nitric oxide synthase expression in A549 cells (Banerjee et al., 2002); reduces tumor nodule formation from B16F-10 melanoma-induced experimental lung metastasis in C57BL/6 mice, with inhibition of the expression of metalloprotease-1 and 2 in lung tissue (Guruvayoorappan and Kuttan, 2007a); induces increase interleukin-2 and interferon-gamma production in Ehrlich ascites carcinoma-bearing BALB/c mice (Guruvayoorappan and Kuttan, 2007b); activates PPARγ/PTEN expressions and induces apoptosis *via* suppressing E7 expression, cell cycle arrest at sub-G1 phase, and mitochondria-emanated intrinsic pathways in SiHa and CaSki cells (Lee et al., 2011); induces apoptosis in MCF-7 cells *via* DNA fragmentation, and de-regulation of intracellular ROS and calcium, alters MMP and activates caspase 3 (Pei et al., 2012); induces anti-angiogenic and anti-metastatic effects through suppression of NF-κB activation in MCF-7 cells (Chen et al., 2015)
Artocarpesin (flavonoid; **14**)	*Morus mesozygia* Stapf (Moraceae) (Kapche et al., 2009; Kuete et al., 2009)	Cytotoxicity toward CCRF-CEM cells, CEM/ADR5000 cells, MDA-MB-231-*pcDNA* cells, MDA-MB-231-*BCRP* cells, HCT116 (*p53^+/+^*) cells, HCT116 (*p53^−/−^*) cells, U87MG cells, U87MG.Δ*EGFR* cells, HepG2 cells (Kuete et al., 2015c)	Hypersensitivity: CEM/ADR5000 cells vs. CCRF-CEM cells (D.R.0.78)(Kuete et al., 2015c)
Atalantoflavone (flavonoid; **15**)	*Erythrina sigmoidea* Hua (Leguminosae) (Kuete et al., 2014c)	Cytotoxicity toward CCRF-CEM cells, CEM/ADR5000 cells, MDA-MB-231-*pcDNA*, HCT116 (*p53^+/+^*), HCT116 (*p53^−/−^*), U87MG, U87MG.Δ*EGFR*, HepG2 (Kuete et al., 2014c)	Hypersensitivity: CEM/ADR5000 cells vs. CCRF-CEM cells (D.R. 0.75); Normal sensitivity: MDA-MB-231-*BCRP* cells vs. MDA-MB-231-*pcDNA* cells (D.R.0.94) (Kuete et al., 2014c)
Bidwillon A (isoflavonoid; **16**)	*Erythrina sigmoidea* Hua (Leguminosae) (Kuete et al., 2014c)	Cytotoxicity toward CCRF-CEM cells, CEM/ADR5000 cells, MDA-MB-231-*BCRP* cells, HCT116 (*p53^+/+^*) cells, HCT116 (*p53^−/−^*) cells, U87MG cells, U87MG.Δ*EGFR* cells, HepG2 cells (Kuete et al., 2014c)	Hypersensitivity: MDA-MB-231-*BCRP* cells vs. MDA-MB-231-*pcDNA* cells (D.R. < 0.17); HCT116 (*p53^−/−^*) cells vs. HCT116 (*p53^+/+^*) cells (D.R. 0.88); U87MG.Δ*EGFR* cells vs. U87MG cells (D.R. 0.39); Normal sensitivity: CEM/ADR5000 cells vs. CCRF-CEM cells (D.R. 1.18) (Kuete et al., 2014c)
Cudraxanthone I (xanthone; **17**)	*Milicia excelsa* Welw C.C. Berg. (Moraceae) (Kuete et al., 2013b)	Cytotoxicity toward CCRF-CEM cells, CEM/ADR5000 cells, MDA-MB-231-*pcDNA* cells, MDA-MB-231-*BCRP* cells, HCT116 (*p53^+/+^*) cells, HCT116 (*p53^−/−^*) cells, U87MG cells, U87MG.Δ*EGFR* cells, HepG2 cells (Kuete et al., 2013b)	Hypersensitivity: CEM/ADR5000 cells vs. CCRF-CEM cells (D.R. 0.78); MDA-MB-231-*BCRP* cells vs. MDA-MB-231-*pcDNA* cells (D.R. 0.36); U87MG.Δ*EGFR* cells vs. U87MG cells (D.R. 0.85); induces apoptosis in CCRF-CEM cells *via* the activation caspases 8 and 9 and caspase 3/7 and loss of MMP (Kuete et al., 2013b)
Cycloartocarpesin (flavonoid; **18**)	*Morus mesozygia* Stapf (Moraceae) (Kapche et al., 2009; Kuete et al., 2009)	Cytotoxicity toward CCRF-CEM cells, CEM/ADR5000 cells, MDA-MB-231-*pcDNA* cells, MDA-MB-231-*BCRP* cells, HCT116 (*p53^+/+^*) cells, HCT116 (*p53^−/−^*) cells, U87MG cells, U87MG.Δ*EGFR* cells, HepG2 cells (Kuete et al., 2015c)	Normal sensitivity: CEM/ADR5000 cells vs. CCRF-CEM cells (D.R.1.04); MDA-MB-231-*BCRP* cells vs. MDA-MB-231-*pcDNA* cells (D.R.1.05); induces apoptosis in CCRF-CEM cells, mediated by caspase 3/7, caspase 8 and 9 activation and the disruption of MMP (Kuete et al., 2015c)
Damnacanthal (anthraquinone; **19**)	*Pentas schimperi* (Hook f.) Verde (Rubiaceae) (Kuete et al., 2015a); *Morinda citrifolia* (Shaghayegh et al., 2016)	Cytotoxicity toward CCRF-CEM cells, CEM/ADR5000 cells, MDA-MB-231-*pcDNA* cells, MDA-MB-231-*BCRP* cells, HCT116 (*p53^+/+^*) cells, HCT116 (*p53^−/−^*) cells, U87MG cells, U87MG.Δ*EGFR* cells, HepG2 cells (Kuete et al., 2015b) (Kuete et al., 2015a)	Hypersensitivity: MDA-MB-231-*BCRP* cells vs. MDA-MB-231-*pcDNA* cells (D.R.0.29); Normal sensitivity: HCT116 (*p53^−/−^*) cells vs. HCT116 (*p53^+/+^*) cells (D.R. 1.05); induces apoptosis and cell cycle arrest in G1 phase, stimulates *p53* and *p21* genes and activates caspase-7 in MCF-7 cells (Aziz et al., 2014); induces apoptosis in CCRF-CEM leukemia cells *via* disruption of the MMP and increase in ROS production (Kuete et al., 2015a); induces apoptosis through inhibition of c-Met in HepG2 cells (García-Vilas et al., 2015); induces apoptosis and cell cycle arrest in oral cancer is H400 oral squamous carcinoma cells through DNA fragmentation and activation of intrinsic apoptosis pathway (Shaghayegh et al., 2016)
Damnacanthol (anthraquinone; **20**)	*Pentas schimperi* (Hook f.) Verde (Rubiaceae) (Kuete et al., 2015a)	Cytotoxicity toward CCRF-CEM cells, CEM/ADR5000 cells, MDA-MB-231-*pcDNA* cells, MDA-MB-231-*BCRP* cells, HCT116 (*p53^+/+^*) cells, HCT116 (*p53^−/−^*) cells, U87MG cells, U87MG.Δ*EGFR* cells, HepG2 cells (Kuete et al., 2015a)	Hypersensitivity: HCT116 (*p53^−/−^*) cells vs. HCT116 (*p53^+/+^*) cells (D.R. 0.77); Normal sensitivity: MDA-MB-231-*BCRP* cells vs. MDA-MB-231-*pcDNA* cells (D.R. 1.09); U87MG.Δ*EGFR* cells vs. U87MG cells (D.R. 1.10); induces apoptosis in CCRF-CEM leukemia cells *via* disruption of the MMP and increase in ROS production (Kuete et al., 2015a)
Dorsmanin F (flavonoid; **21**)	*Dorstenia mannii* Hook.f. (Moraceae) (Ngadjui et al., 1999a)	Cytotoxicity toward CCRF-CEM cells, CEM/ADR5000 cells, MDA-MB-231-*pcDNA* cells, MDA-MB-231-*BCRP* cells, HCT116 (*p53^+/+^*) cells, HCT116 (*p53^−/−^*) cells, U87MG cells, U87MG.Δ*EGFR* cells, HepG2 cells (Kuete et al., 2015d)	Hypersensitivity: U87MG.Δ*EGFR* cells vs. U87MG cells (D.R. 0.61); induces apoptosis in CCRF-CEM cells *via* the disruption of MMP (Kuete et al., 2015d)
Euxanthone (xanthone; **22**)	*Oricia suaveolens* Engl. (Rutaceae) (Fouotsa et al., 2013)	Cytotoxicity toward CCRF-CEM cells, CEM/ADR5000 cells, MDA-MB-231-*pcDNA* cells, MDA-MB-231-*BCRP* cells, HCT116 (*p53^+/+^*) cells, HCT116 (*p53^−/−^*) cells, U87MG cells, U87MG.Δ*EGFR* cells, HepG2 cells (Kuete et al., 2016c); HeLa cells, CEM-SS cells and CaOV3 cells (Ee et al., 2005)	Hypersensitivity: MDA-MB-231-*BCRP* cells vs. MDA-MB-231-*pcDNA* cells (D.R.0. < 0.07); HCT116 (*p53^−/−^*) cells vs. HCT116 (*p53^+/+^*) cells (D.R. 0.65); Normal sensitivity: U87MG.Δ*EGFR* cells vs. U87MG cells (D.R. 1.17)(Kuete et al., 2016c)
Futokadsurin B (lignan; **23**)	*Uapaca togoensis* Pax (Euphorbiaceae) (Kuete et al., 2015e)	Cytotoxicity toward CCRF-CEM cells, CEM/ADR5000 cells, MDA-MB-231-*pcDNA* cells, MDA-MB-231-*BCRP* cells, HCT116 (*p53^−/−^*) cells, HepG2 cells (Kuete et al., 2015e)	Hypersensitivity: CEM/ADR5000 cells vs. CCRF-CEM cells (D.R.0.36); HCT116 (*p53^−/−^*) cells vs. HCT116 (*p53^+/+^*) cells (D.R. < 0.37) (Kuete et al., 2016c); Normal sensitivity: MDA-MB-231-*BCRP* cells vs. MDA-MB-231-*pcDNA* cells (D.R.1.14) (Kuete et al., 2015e)
Gancaonin Q (flavonoid; **24**)	*Dorstenia angusticornis* Engl. (Kuete et al., 2007b)	Cytotoxicity toward CCRF-CEM cells, CEM/ADR5000 cells, PF-382 cells, HL-60 cells, MiaPaCa-2 cells, Capan-1 cells, MCF-7 cells, SW-680 cells, 786-0 cells, U87MG cells, A549 cells, Colo-38 cells, HeLa cells, Caski cells (Kuete et al., 2011b)	Induces apoptosis in CCRF-CEM cells *via* caspase 3/7 activation; anti-angiogenic effect *via* inhibition of the growth of blood capillaries on the chorioallantoic membrane of quail eggs (Kuete et al., 2011b)
Guieranone A (naphthyl butenone; **25**)	*Guiera senegalensis* J. F. Gmel. (Combretaceae) (Kuete et al., 2012)	Cytotoxicity toward CCRF-CEM cells, CEM/ADR5000 cells, PF-382 cells, MiaPaCa-2 cells, Capan-1 cells, MCF-7 cells, 786-0 cells, U87MG cells, A549 cells, Colo-38 cells, HeLa cells, Caski cells (Kuete et al., 2012), THP-1 cells (Fiot et al., 2006)	Showed anti-angiogenic activity *via* the inhibition of the growth of blood capillaries on the chorioallantoic membrane of quail embryo; induces apoptosis in CCRF-CEM and cell cycle arrest; affects the regulation of several pathways in CCRF-CEM cells such as the Cell Cycle: G2/M DNA Damage Checkpoint Regulation and ATM Signaling pathways (Kuete et al., 2012)
Guttiferone E (benzophenone; **26**)	*Garcinia punctata Oliv*.(Guttiferae) (Kuete et al., 2013d)	Cytotoxicity toward CCRF-CEM cells, CEM/ADR5000 cells, MDA-MB-231-*pcDNA* cells, MDA-MB-231-*BCRP* cells, HCT116 (*p53^+/+^*) cells, HCT116 (*p53^−/−^*) cells, U87MG cells, U87MG.Δ*EGFR* cells, HepG2 cells (Kuete et al., 2013d); HT29 cells (Einbond et al., 2013) and SW-480 (Baggett et al., 2005; Protiva et al., 2008)	Hypersensitivity: HCT116 (*p53^−/−^*) cells vs. HCT116 (*p53^+/+^*) cells (D.R. 0.62); U87MG.Δ*EGFR* cells vs. U87MG cells (D.R. 0.43); Normal sensitivity: HL60AR cells vs. HL60 cells (D.R. 1.00); MDA-MB-231-*BCRP* cells vs. MDA-MB-231-*pcDNA* cells (D.R.1.19); induces apoptosis in HCT116, HT29 and SW480 cells though loss MMP and caspase 3/7 activation (Protiva et al., 2008); induces apoptosis in CCRF-CEM cells *via* activation of inititator caspases 8 and 9 and effector caspase 3/7 as well as loss of MMP (Kuete et al., 2013d); induces apoptosis in HeLa cells (Liu et al., 2010)
Isobavachalcone (flavonoid; **27**)	*Dorstenia barteri* Bureau *var. multiradiata* (Moraceae) (Mbaveng et al., 2008; Kuete and Sandjo, 2012)	Cytotoxicity toward CCRF-CEM cells, CEM/ADR5000 cells, MDA-MB-231-*pcDNA* cells, MDA-MB-231-*BCRP* cells, HCT116 (*p53^+/+^*) cells, HCT116 (*p53^−/−^*) cells, U87MG cells, U87MG.Δ*EGFR* cells, HepG2 cells (Kuete et al., 2015c); OVCAR-8 cells, MCF-7 cells and A549 cells (Jing et al., 2010; Kuete and Sandjo, 2012).	Hypersensitivity: MDA-MB-231-*BCRP* cells vs. MDA-MB-231-*pcDNA* cells (D.R.0.13); HCT116 (*p53^−/−^*) cells vs. HCT116 (*p53^+/+^*) cells (D.R. 0.84); U87MG.Δ*EGFR* cells vs. U87MG cells (D.R. 0.73); induces apoptosis in IMR-32 and NB-39 cells *via* activation of caspase-3 and -9 and and Bax upregulation (Nishimura et al., 2007); inhibits matrix metalloproteinases-2 secretion in U87 cells (Ngameni et al., 2007); induces apoptosis in CCRF-CEM cells, mediated by caspase 3/7, 8 and 9 activation, the disruption of MMP and increase ROS production (Kuete et al., 2015c)
Isogarcinol (benzophenone; **28**)	*Hypericum lanceolatum Lam*. (Hypericaceae) (Kuete et al., 2013d; Pieme et al., 2015). *Garcinia ovalifolia* (Pieme et al., 2015)	Cytotoxicity toward CCRF-CEM cells, CEM/ADR5000 cells, MDA-MB-231-*pcDNA* cells, MDA-MB-231-*BCRP* cells, HCT116 (*p53^+/+^*) cells, HCT116 (*p53^−/−^*) cells, U87MG cells, U87MG.Δ*EGFR* cells, HepG2 cells (Kuete et al., 2013d); HL60 cells (Pieme et al., 2015)	Hypersensitivity: MDA-MB-231-*BCRP* cells vs. MDA-MB-231-*pcDNA* cells (D.R.0.31); U87MG.Δ*EGFR* cells vs. U87MG cells (D.R. 0.83); Normal sensitivity: HL60AR cells vs. HL60 cells (D.R. 1.02) (Kuete et al., 2013d); induces G2/S cycle arrest and apoptosis in HL60 cells through MMP loss (Pieme et al., 2015)
Isoneorautenol (isoflavonoid; **29**)	*Erythrina excelsa* Baker (Fabaceae) (Kuete et al., 2014d)	Cytotoxicity toward CCRF-CEM cells, CEM/ADR5000 cells, MDA-MB-231-*pcDNA* cells, MDA-MB-231-*BCRP* cells, HCT116 (*p53^+/+^*) cells, HCT116 (*p53^−/−^*) cells, U87MG cells, U87MG.Δ*EGFR* cells, HepG2 cells (Kuete et al., 2014d); H4IIE cells (Watjen et al., 2007).	Hypersensitivity: MDA-MB-231-*BCRP* cells vs. MDA-MB-231-*pcDNA* cells (D.R.0.17); HCT116 (*p53^−/−^*) cells vs. HCT116 (*p53^+/+^*) cells (D.R. 0.78); Normal sensitivity: U87MG.Δ*EGFR* cells vs. U87MG cells (D.R. 0.93); induces apoptosis in CCRF-CEM cells *via* the activation of caspases 8 and 9 and caspase 3/7; loss of MMP and increase ROS production (Kuete et al., 2014d)
Isoxanthochymol (benzophenone; **30**)	*Garcinia punctata Oliv*.(Guttiferae) (Kuete et al., 2013d)	Cytotoxicity toward CCRF-CEM cells, CEM/ADR5000 cells, MDA-MB-231-*pcDNA* cells, MDA-MB-231-*BCRP* cells, HCT116 (*p53^+/+^*) cells, HCT116 (*p53^−/−^*) cells, U87MG cells, U87MG.Δ*EGFR* cells, HepG2 cells (Kuete et al., 2013d) and SW-480 (Baggett et al., 2005)	Hypersensitivity: MDA-MB-231-*BCRP* cells vs. MDA-MB-231-*pcDNA* cells (D.R.0.54); U87MG.Δ*EGFR* cells vs. U87MG cells (D.R. 0.49); Normal sensitivity: CEM/ADR5000 cells vs. CCRF-CEM cells (D.R. 1.08); HL60AR cells vs. HL60 cells (D.R. 1.00); HCT116 (*p53^−/−^*) cells vs. HCT116 (*p53^+/+^*) cells (D.R. 1.12); induces apoptosis in CCRF-CEM cells *via* activation of inititator caspases 8 and 9 and effector caspase 3/7 as well as loss of MMP (Kuete et al., 2013d); induces apoptosis in HeLa cells (Liu et al., 2010).
Laburnetin (flavonoid; **32**)	*Ficus chlamydocarpa* Mildbr. & Burret (Moraceae) (Kuete et al., 2008)	Cytotoxicity toward CCRF-CEM cells, CEM/ADR5000 cells, MDA-MB-231-*pcDNA* cells, MDA-MB-231-*BCRP* cells, HCT116 (*p53^+/+^*) cells, HCT116 (*p53^−/−^*) cells, U87MG cells, U87MG.Δ*EGFR* cells, HepG2 cells (Kuete et al., 2016c); UMR106 cells (Xiaoli et al., 2006)	Hypersensitivity: HCT116 (*p53^−/−^*) cells vs. HCT116 (*p53^+/+^*) cells (D.R. 0.74); U87MG.Δ*EGFR* cells vs. U87MG cells (D.R. 0.86) (Kuete et al., 2016c); Normal sensitivity: MDA-MB-231-*BCRP* cells vs. MDA-MB-231-*pcDNA* cells (D.R.0.98) (Kuete et al., 2016c)
Morusignin I (xanthone; **33**)	*Garcinia nobilis* Engl. (Guttiferae) (Fouotsa et al., 2012, 2013)	Cytotoxicity toward CCRF-CEM cells, CEM/ADR5000 cells, MDA-MB-231-*pcDNA* cells, MDA-MB-231-*BCRP* cells, HCT116 (*p53^+/+^*) cells, HCT116 (*p53^−/−^*) cells, U87MG cells, U87MG.Δ*EGFR* cells, HepG2 cells (Kuete et al., 2013b)	Normal sensitivity: MDA-MB-231-*BCRP* cells vs. MDA-MB-231-*pcDNA* cells (D.R. 1.03) (Kuete et al., 2013b)
Naringenin (flavonoid; **34**)	*Aframomum arundinaceum (Oliver & Hanbury) K. Schum* (Zinziberaceae)(Kuete et al., 2014a)	Cytotoxicity toward CCRF-CEM cells, CEM/ADR5000 cells, MDA-MB-231-*pcDNA* cells, MDA-MB-231-*BCRP* cells, HCT116 (*p53^+/+^*) cells, HCT116 (*p53^−/−^*) cells, U87MG cells, U87MG.Δ*EGFR* cells, HepG2 cells (Kuete et al., 2014a)	Hypersensitivity: CEM/ADR5000 cells vs. CCRF-CEM cells (D.R. 0.64); U87MG.Δ*EGFR* cells vs. U87MG cells (D.R. 0.60); HepG2 cells vs. AML12 cells (D.R. < 0.59); Normal sensitivity: HCT116 (*p53^−/−^*) cells vs. HCT116 cells (*p53^+/+^*) (D.R. 1.02)(Kuete et al., 2014a); induces apoptosis in HepG2 cells and DLD-1 cells through caspase-3 activation and poly(ADP-ribose) polymerase cleavage (Totta et al., 2004); induces apoptosis in HL60 cells through activation of NF-kappaB and necrosis involving the loss of ATP (Kanno et al., 2006); induces apoptosis in THP-1 cells through downregulation of Akt and caspase-3 activation (Park et al., 2008); up-regulates the expression of death receptor 5 and enhances TRAIL-induced apoptosis in A549 cells (Jin et al., 2011)
Neobavaisoflavone (isoflavonoid; **35**)	*Erythrina senegalensis DC* (Fabaceae) (Kuete et al., 2014d)	Cytotoxicity toward CCRF-CEM cells, CEM/ADR5000 cells, HCT116 (*p53^+/+^*) cells, HCT116 (*p53^−/−^*) cells, U87MG.Δ*EGFR* cells, HepG2 cells (Kuete et al., 2014d); LNCaP cells (Szliszka et al., 2011).	Hypersensitivity: HCT116 (*p53^−/−^*) cells vs. HCT116 (*p53^+/+^*) cells (D.R. 0.87); U87MG.Δ*EGFR* cells vs. U87MG cells (D.R. < 0.58); Normal sensitivity: CEM/ADR5000 cells vs. CCRF-CEM cells (D.R.1.20) (Kuete et al., 2014d); induces apoptosis *via* the inhibition of metastasis in U373MG cells (Kim et al., 2014); induces TRAIL-mediated apoptosis in LNCaP cells (Szliszka et al., 2011)
Neocyclomorusin (flavonoid; **36**)	*Erythrina sigmoidea* Hua (Leguminosae) (Kuete et al., 2014c)	Cytotoxicity toward CCRF-CEM cells, CEM/ADR5000 cells, MDA-MB-231-*pcDNA* cells, HCT116 (*p53^+/+^*) cells, U87MG cells, U87MG.Δ*EGFR* cells (Kuete et al., 2014c)	Normal sensitivity: CEM/ADR5000 cells vs. CCRF-CEM cells (D.R. 1.19) (Kuete et al., 2014c)
Plumbagin (naphthoquinone; **37**)	*Plumbago dawei* (Maniafu et al., 2009); *Plumbago zeylanica*; (Kuete et al., 2016d); *Diospyros crassiflora* and *Diospyros canaliculata* (Kuete and Efferth, 2011)	Cytotoxicity toward A549 cells, SPC212 cells; DLD-1 cells, Caco2 cells; MCF-7 cells (Kuete et al., 2016d)	Induces ROS-mediated apoptosis, release of mitochondrial cytochrome c and activation of caspase-3 and -9 in ME-180 cells (Srinivas et al., 2004); induces apoptosis in MCF-7 cells mediated by increase ROS production and MMP loss (Kuete et al., 2016d); DNA: Methylation of CpG site(s) – 47.48.49 – in ARHGEF12 (Kuete and Efferth, 2011); inhibits the NF-kappaB activation pathway in cancer cells leading to suppression of NF-kappaB-regulated gene products (Sandur et al., 2006); induces apoptosis and cell cycle arrest in A549 cells through *p53* accumulation *via c-*Jun NH2-terminal kinase-mediated phosphorylation (Hsu et al., 2006); induces apoptosis in MDA-MB-231 and MCF-7 cells *via* inactivation of NF-κB and Bcl-2 (Ahmad et al., 2008); induces apoptosis in PC-3 cells, LNCaP cells and C4-2 cells through ROS generation, depletion of intracellular glutathione levels; reduces expression of superoxide dismutase 2 (Powolny and Singh, 2008); induces ROS-mediated apoptosis in NB4 cells *in vivo* (Xu and Lu, 2010)
Poinsettifolin B (flavonoid; **38**)	*Dorstenia poinsettifolia* Engl. (Moraceae) (Ngadjui et al., 1999b)	Cytotoxicity toward CCRF-CEM cells, CEM/ADR5000 cells, MDA-MB-231-*pcDNA* cells, MDA-MB-231-*BCRP* cells, HCT116 (*p53^+/+^*) cells, HCT116 (*p53^−/−^*) cells, U87MG cells, U87MG.Δ*EGFR* cells, HepG2 cells (Kuete et al., 2015d)	Hypersensitivity: HCT116 (*p53^−/−^*) cells vs. HCT116 (*p53^+/+^*) cells (D.R. 0.64); Normal sensitivity: MDA-MB-231-*BCRP* cells vs. MDA-MB-231-*pcDNA* cells (D.R.1.19); induces apoptosis in CCRF-CEM cells *via* the disruption of MMP and increase ROS production (Kuete et al., 2015d)
Pycnanthulignene A (lignan; **39**)	*Pycnanthus angolensis* (Welw.) Ward (Myristicaceae) (Nono et al., 2010; Kuete et al., 2011c)	Cytotoxicity toward CCRF-CEM cells, CEM/ADR5000 cells, MDA-MB-231-*pcDNA* cells, MDA-MB-231-*BCRP* cells, HCT116 (*p53^+/+^*) cells, HCT116 (*p53^−/−^*) cells, U87MG cells, U87MG.Δ*EGFR* cells, HepG2 cells (Kuete et al., 2016c)	Hypersensitivity: CEM/ADR5000 cells vs. CCRF-CEM cells (D.R.0.78); MDA-MB-231-*BCRP* cells vs. MDA-MB-231-*pcDNA* cells (D.R.0.88); Normal sensitivity: U87MG.Δ*EGFR* cells vs. U87MG cells (D.R. 1.02); induces apoptosis in CCRF-CEM cells, mediated by loss of MMP and increase ROS production (Kuete et al., 2016c)
Pycnanthulignene B (lignan; **40**)	*Pycnanthus angolensis* (Welw.) Ward (Myristicaceae) (Nono et al., 2010; Kuete et al., 2011c)	Cytotoxicity toward CEM/ADR5000 cells (Kuete et al., 2016c)	Hypersensitivity: CEM/ADR5000 cells vs. CCRF-CEM cells (D.R. < 0.14) (Kuete et al., 2016c)
Kaempferol-3,7,4′-trimethylether (flavonoid; **31**)	*Aframomum arundinaceum (Oliver & Hanbury) K. Schum* (Zinziberaceae)(Kuete et al., 2014a)	Cytotoxicity toward CCRF-CEM cells, CEM/ADR5000 cells, MDA-MB-231-*BCRP* cells, HCT116 (*p53^−/−^*) cells, U87MG cells, U87MG.Δ*EGFR* cells (Kuete et al., 2014a)	Hypersensitivity: MDA-MB-231-*BCRP* cells vs. MDA-MB-231-*pcDNA* cells (D.R. < 0.83); HCT116 (*p53^−/−^*) cells vs. HCT116 (*p53^+/+^*) cells (D.R. < 0.82); CEM/ADR5000 cells vs. CCRF-CEM cells (D.R. 0.99) (Kuete et al., 2014a)
Rapanone (benzoquinone; **41**)	*Maesa lanceolata* Forssk., *Myrsine Africana* L., *Embelia keniensis* R.E.Fr., *Embelia schimperi Vatke* and *Rapanea pulchra* Gilg & Schellenb. (Ogweno Midiwo et al., 2002)	Cytotoxicity toward A549 cells, SPC212 cells; DLD-1, Caco2 cells; MCF-7 cells (Kuete et al., 2016d)	Induces apoptosis in MCF-7 cells mediated by increase ROS production and MMP loss (Kuete et al., 2016d)
Resveratrol *β-*_D_*-*glucopyranoside (stilbene; **42**)	*Nauclea pobeguinii* (Pobég. ex Pellegr.) Merr. ex E.M.A. (Rubiaceae) (Kuete et al., 2015f)	Cytotoxicity toward CCRF-CEM cells, CEM/ADR5000 cells, MDA-MB-231-*pcDNA* cells, MDA-MB-231-*BCRP* cells, HCT116 (*p53^+/+^*) cells, HCT116 (*p53^−/−^*) cells, U87MG cells, U87MG.Δ*EGFR* cells, HepG2 cells (Kuete et al., 2015f)	Hypersensitivity: HCT116 (*p53^−/−^*) cells vs. HCT116 (*p53^+/+^*) cells (D.R. 0.74); U87MG.Δ*EGFR* cells vs. U87MG cells (D.R. 0.86); Normal sensitivity: MDA-MB-231-*BCRP* cells vs. MDA-MB-231-*pcDNA* cells (D.R.0.98) (Kuete et al., 2015f)
Sigmoidin H (isoflavonoid; **43**)	*Erythrina senegalensis DC* (Fabaceae) (Kuete et al., 2014d)	Cytotoxicity toward CCRF-CEM cells, CEM/ADR5000 cells, HCT116 (*p53^+/+^*) cells, U87MG cells (Kuete et al., 2014d)	Normal sensitivity: CEM/ADR5000 cells vs. CCRF-CEM cells (D.R.1.02) (Kuete et al., 2014d)
Sigmoidin I (isoflavonoid; **44**)	*Erythrina sigmoidea* Hua (Leguminosae) (Kuete et al., 2014c)	Cytotoxicity toward CCRF-CEM cells, CEM/ADR5000 cells, MDA-MB-231-*pcDNA* cells, MDA-MB-231-*BCRP* cells, HCT116 (*p53^+/+^*) cells, U87MG.Δ*EGFR* cells, HepG2 cells (Kuete et al., 2014c)	Hypersensitivity: HCT116 (*p53^−/−^*) cells vs. HCT116 (*p53^+/+^*) cells (D.R. 87); U87MG.Δ*EGFR* cells vs. U87MG cells (D.R. 0.65); induces apoptosis in CCRF-CEM cells *via* breakdown of MMP and increase in ROS production (Kuete et al., 2014c)
Sophorapterocarpan A (isoflavonoid; **45**)	*Erythrina sigmoidea* Hua (Leguminosae) (Kuete et al., 2014c)	Cytotoxicity toward CCRF-CEM cells, CEM/ADR5000 cells, MDA-MB-231-*pcDNA* cells, MDA-MB-231-*BCRP* cells, HCT116 (*p53^+/+^*) cells, HCT116 (*p53^−/−^*) cells, U87MG cells, U87MG.Δ*EGFR* cells, HepG2 cells (Kuete et al., 2014c)	Hypersensitivity: MDA-MB-231-*BCRP* cells vs. MDA-MB-231-*pcDNA* cells (D.R.0.90); U87MG.Δ*EGFR* cells vs. U87MG cells (D.R. 0.69); Normal sensitivity: HCT116 (*p53^−/−^*) cells vs. HCT116 (*p53^+/+^*) cells (D.R. 0.95); induces apoptosis in CCRF-CEM cells *via* breakdown of MMP and increase in ROS production (Kuete et al., 2014c)
Xanthone V_1_ (xanthone; **46**)	*Vismia laurentii* De Wild. (Guttiferae) (Wabo et al., 2007)	Cytotoxicity toward CCRF-CEM cells, CEM/ADR5000 cells, HL-60 cells, MiaPaCa-2 cells, MCF-7 cells, SW-680 cells, 786-0 cells, U87MG cells, A549 cells, Colo-38 cells, HeLa cells, Caski cells (Kuete et al., 2011d)	Anti-angiogenic effects through inhibition of the growth of blood capillaries on the chorioallantoic membrane of quail eggs; induces apoptosis and cell cycle arrest in S-phase in CCRF-CEM cells mediated by Caspase 3/7 activation (Kuete et al., 2011d)
**ALKALOIDS**			
1,3-dimethoxy-10-methylacridone (acridone; **47**)	*Oricia suaveolens* Engl. (Rutaceae) (Fouotsa et al., 2013)	Cytotoxicity toward CCRF-CEM cells, CEM/ADR5000 cells, MDA-MB-231-*pcDNA* cells, MDA-MB-231-*BCRP* cells, HCT116 (*p53^+/+^*) cells, HCT116 (*p53^−/−^*) cells, U87MG cells, U87MG.Δ*EGFR* cells, HepG2 cells (Kuete et al., 2015b)	Hypersensitivity: MDA-MB-231-*BCRP* cells vs. MDA-MB-231-*pcDNA* cells (D.R. 0.33); U87MG.Δ*EGFR* cells vs. U87MG cells (D.R. 0.51); Normal sensitivity: HCT116 (*p53^−/−^*) cells vs. HCT116 (*p53^+/+^*) cells (D.R. 0.97); induces apoptosis in CCRF-CEM cells, mediated by ROS production. (Kuete et al., 2015b)
1-hydroxy-3-methoxy-10-methylacridone (acridone; **48**)	*Oricia suaveolens* Engl. (Rutaceae) (Fouotsa et al., 2013)	Cytotoxicity toward CCRF-CEM cells, CEM/ADR5000 cells, MDA-MB-231-*pcDNA* cells, MDA-MB-231-*BCRP* cells, HCT116 (*p53^+/+^*) cells, HCT116 (*p53^−/−^*) cells, U87MG cells, U87MG.Δ*EGFR* cells, HepG2 cells (Kuete et al., 2015b)	Hypersensitivity: MDA-MB-231-*BCRP* cells vs. MDA-MB-231-*pcDNA* cells (D.R. 0.18); HCT116 (*p53^−/−^*) cells vs. HCT116 (*p53^+/+^*) cells (D.R. 0.39); U87MG.Δ*EGFR* cells vs. U87MG cells (D.R. 0.21) (Kuete et al., 2015b)
Arborinine (acridone; **49**)	*Uapaca togoensis* Pax (Euphorbiaceae) (Kuete et al., 2015e)	Cytotoxicity toward CCRF-CEM cells, CEM/ADR5000 cells, MDA-MB-231-*pcDNA* cells, MDA-MB-231-*BCRP* cells, HCT116 (*p53^+/+^*) cells, HCT116 (*p53^−/−^*) cells, U87MG cells, U87MG.Δ*EGFR* cells, HepG2 cells (Kuete et al., 2015e)	Hypersensitivity: CEM/ADR5000 cells vs. CCRF-CEM cells (D.R. 0.11); MDA-MB-231-*BCRP* cells vs. MDA-MB-231-*pcDNA* cells (D.R. 0.87); U87MG.Δ*EGFR* cells vs. U87MG cells (D.R. 0.34); induces apoptosis in CCRF-CEM cells (Kuete et al., 2015e)
Evoxanthine (acridone; **50**)	*Oricia suaveolens* Engl. (Rutaceae) (Fouotsa et al., 2013)	Cytotoxicity toward CCRF-CEM cells, CEM/ADR5000 cells, MDA-MB-231-*pcDNA* cells, MDA-MB-231-*BCRP* cells, HCT116 (*p53^+/+^*) cells, HCT116 (*p53^−/−^*) cells, U87MG cells, U87MG.Δ*EGFR* cells, HepG2 cells (Kuete et al., 2015b)	Hypersensitivity: U87MG.Δ*EGFR* cells vs. U87MG cells (D.R. 0.45) (Kuete et al., 2015b)
Isotetrandrine (isoquinoline; **51**)	*Xylopia aethiopica* (Dunal) A.Rich. (Annonaceae) (Kuete et al., 2015g)	Cytotoxicity toward CCRF-CEM cells, MDA-MB-231-*pcDNA* cells, MDA-MB-231-*BCRP* cells, HCT116 (*p53^+/+^*) cells, HCT116 (*p53^−/−^*) cells, U87MG cells, U87MG.Δ*EGFR* cells, HepG2 cells (Kuete et al., 2015g)	Hypersensitivity: U87MG.Δ*EGFR* cells vs. U87MG cells (D.R. 0.38); Normal sensitivity: MDA-MB-231-*BCRP* cells vs. MDA-MB-231-*pcDNA* cells (D.R. 0.92); induces apoptosis in CCRF-CEM cells, mediated byROS production (Kuete et al., 2015g)
Montrifoline (**52**)	*Oricia suaveolens* Engl. (Rutaceae) (Fouotsa et al., 2013)	Cytotoxicity toward CCRF-CEM cells, CEM/ADR5000 cells, MDA-MB-231-*pcDNA* cells, MDA-MB-231-*BCRP* cells, HCT116 (*p53^+/+^*) cells, HCT116 (*p53^−/−^*) cells, U87MG cells, U87MG.Δ*EGFR* cells, HepG2 cells (Kuete et al., 2015b)	Hypersensitivity: U87MG.Δ*EGFR* cells vs. U87MG cells (D.R. 0.74) (Kuete et al., 2015b)
Norevoxanthine (acridone; **53**)	*Oricia suaveolens* Engl. (Rutaceae) (Fouotsa et al., 2013)	Cytotoxicity toward CCRF-CEM cells, CEM/ADR5000 cells, MDA-MB-231-*pcDNA*, MDA-MB-231-*BCRP*, HCT116 (*p53^+/+^*), HCT116 (*p53^−/−^*), U87MG, U87MG.Δ*EGFR*, HepG2 (Kuete et al., 2015b)	Hypersensitivity: CEM/ADR5000 cells vs. CCRF-CEM cells (D.R. 0.75); U87MG.Δ*EGFR* cells vs. U87MG cells (D.R. 0.06); Normal sensitivity: MDA-MB-231-*BCRP* cells vs. MDA-MB-231-*pcDNA* cells (D.R. 1.17) (Kuete et al., 2015b)

a *Reported cell lines: leukemia cells [CCRF-CEM, CEM/ADR5000, HL60, and HL60AR; NB4 cells; PF-382 cells; CEM-SS cells, THP-1 cells]; Carcinoma cells [MDA-MB-231-pcDNA3, MDA-MB-231-BCRP clone 23, and MCF-7 breast cancer cells, HT29, SW-480, SW-680, HCT116 (p53^+/+^), and HCT116 (p53^−/−^) colon cancer cells, U373MG, U87MG, and U87MG.ΔEGFR glioblastoma cells; HepG2: hepatocarcinoma; IMR-32 and NB-39 human neuroblastoma cells; AML12: normal hepatocytes; A549 human non-small cell lung cancer (NSCLC) cells, SPC212 human mesothelioma cells; H2108 and H1299 lung cancer cells; DLD-1 and Caco2 colorectal adenocarcinoma cells; CaOV3 and OVCAR-8 ovarian carcinoma cells; C4-2, MiaPaca-2, Capan-1 and PC-3 prancreatic carcinoma cells, LNCaP human prostatic adenocarcinoma, 786-0 renal carcinoma cells; ME-180, SiHa, Caski and HeLa cervical carcinoma cells, B16F-10 and Colo-38 skin melanoma cells; H4IIE rat hepatoma cells; UMR106 rat osteogenic sarcoma cells]; D.R., degree of resistance; D.R. is determined as the ratio of IC_50_ value in the resistant divided by the IC_50_ in the sensitive cell line; AML12, HL60AR, CEM/ADR5000, MDA-MB-231-BCRP, HCT116 (p53^−/−^) and U87MG. ΔEGFR were used as the corresponding resistant counterpart for HepG2, HL60, CCRF-CEM, MDA-MB-231-pcDNA, HCT116 (p53^+/+^), U87MG, respectively; Hypersensitivity, D.R. < 1; Normal sensitivity, D.R. equal of around 1; MMP, mitochondrial membrane potential; ROS, reactive oxygen species*.

### Plants and derived compounds targeting the mitochondria of cancer cells

Several crude extracts and isolated compounds from CEWA plants targeted mitochondria to induce apoptosis in cancer cells (Tables 1, 2). Plant extracts inducing MMP alterations include: the Annonaceae plants *Annona muricata* Lin. (Kuete et al., 2016b), *Anonidium mannii* (oliv) Engl. et Diels. (Kuete et al., 2013a) and *Xylopia aethiopica* (Dunal) A. Rich. (Kuete et al., 2013c), a plant of Araliaceae family, *Polyscias fulva* (Hiern) Harms. (Kuete et al., 2014e), of Bignoniaceae, *Markhamia tomentosa* (Benth.) K.Schumex.Engl. (Ibrahim et al., 2013), of Compositae, *Echinops giganteus* var. lelyi (C. D. Adams) A. Rich. (Kuete et al., 2013c), the Euphorbiaceae, *Alchornea cordifolia* (Schum. & Thonn.) Müll.-Arg. (Kuete et al., 2016e) and *Uapaca togoensis* Pax. (Kuete et al., 2015e), the Fabaceae, *Albizia adianthifolia* (Schum.) (Kuete et al., 2016e) and *Erythrina sigmoidea* Hua (Kuete et al., 2016a), the Moraceae, *Dorstenia psilurus* Welwitch (Pieme et al., 2013), the Passifloraceae, *Passiflora edulis* Sims (Kuete et al., 2016b), the Piperaceae, *Piper capense* L.f. (Kuete et al., 2013c), the Poaceae, *Imperata cylindrica* Beauv. var. koenigii Durand et Schinz (Kuete et al., 2013c) and the Rutaceae, *Vepris soyauxii* Engl. (Kuete et al., 2013a). Several molecules isolated from plants of CEWA also targeted mitochondria. Some of them include: anthraquinones, damnacanthal (**19**) and damnacanthol (**20**) from *Pentas schimperi* (Hook f.) Verde (Kuete et al., 2015a), the benzoquinone, rapanone (**41**) from *Maesa lanceolata* Forssk., *Myrsine africana* L., *Embelia keniensis* R.E.Fr., *Embelia schimperi* Vatke and *Rapanea pulchra* Gilg & Schellenb. and the naphthoquinone, plumbagin (**37**) from *Plumbago* and *Diospyros* species (Kuete et al., 2016d), benzophenones, **26** and **30** (Kuete et al., 2013d), flavonoids, 3,4′,5-trihydroxy-6″,6″-dimethylpyrano[2,3-g]flavone (**5**) isolated *Xylopia aethiopica* (Dunal) A.Rich. (Kuete et al., 2015g), 4′-hydroxy-2′,6′-dimethoxychalcone (**6**) from *Polygonum limbatum* Meisn (Kuete et al., 2014b), abyssinone IV (**11**) from *Erythrina sigmoidea* Hua (Kuete et al., 2014c), alpinumisoflavone (**12**) from *Ficus chlamydocarpa* Mildbr. & Burret (Kuete et al., 2014c), dorsmanin F (**21**) from *Dorstenia mannii* Hook.f. (Kuete et al., 2015d), compound **27** (Kuete et al., 2015c) and poinsettifolin B (**38**) from *Dorstenia poinsettifolia* Engl. (Kuete et al., 2015d), isoflavonoids, 6α-hydroxyphaseollidin (**9**) (Kuete et al., 2014c), isoneorautenol (**29**) (Kuete et al., 2014d), sigmoidin I (**44**) and sophorapterocarpan A (**45**) (Kuete et al., 2014c) from *Erythrina sigmoidea* Hua, a lignan, pycnanthulignene A (**39**) from *Pycnanthus angolensis* (Welw.) Ward (Kuete et al., 2016c) and a xanthone, cudraxanthone I (**17**) from *Milicia excelsa* Welw C.C. Berg. (Kuete et al., 2013b).

### Plants and derived compounds inducing ROS increase in cancer cells

The induction of apoptosis in cancer cells by some plants and derived molecules of CEWA was due to induced-ROS increase (Tables 1, 2). Plants that exert their anticancer activities via this mechanism include: *Alchornea cordifolia* (Schum. & Thonn.) Müll.-Arg. (Kuete et al., 2016e), *Anonidium mannii* (oliv) Engl. et Diels. (Kuete et al., 2013a), *Dorstenia psilurus* Welwitch (Pieme et al., 2013), *Piper capense* L.f. (Kuete et al., 2013c), *Polyscias fulva* (Hiern) Harms. (Kuete et al., 2014e), *Sclerocarya birrea* (A. Rich.) Hochst (Anacardiaceae) (Armentano et al., 2015). Compounds inducing increase in ROS production include: acridone alkaloid 1,3-dimethoxy-10-methylacridone (**47**) and isoquinoline alkaloid isotetrandrine (**51**), anthraquinones **19** and **20** (Kuete et al., 2015a), benzoquinone **41** (Kuete et al., 2016d) and naphthoquinone **37** (Kuete et al., 2016d), flavonoids **6** (Kuete et al., 2014b), **11** (Kuete et al., 2014c), **12** (Kuete et al., 2016c), **27** (Kuete et al., 2015c), **38** (Kuete et al., 2015d), isoflavonoids **9** (Kuete et al., 2014c), **29** (Kuete et al., 2014d), **44** and **45** (Kuete et al., 2014c), and lignan **39** (Kuete et al., 2016c).

### Angiogenesis

Angiogenesis as treatment target against cancer was merely studied for botanicals from Africa. The Fabaceae *Calliandra portoricensis* (Jacq.) (Adaramoye et al., 2015) was reported as angiogenesis inhibitor through inhibition of the growth of blood capillaries on the chorioallantoic membrane of quail eggs. Compounds with similar effects include naphthoquinone 2-acetylfuro-1,4-naphthoquinone (**4**) (Kuete et al., 2011d), flavonoids **7**, **8**, and **24** (Kuete et al., 2011b), the naphthyl butenone guieranone A (**25**) and the xanthone **46** (Kuete et al., 2011d).

### African plants and compounds with regular sensitivity and collateral sensitivity in drug resistant cancer cells

The investigation of the mode of action of botanicals and phytochemicals from the flora of Africa is not yet done in a systematic manner due to the lack of facilities and appropriate technology in research centers throughout the continent. However, the fight against MDR in cancer will provide conceptual clues on the molecular targets of the active samples. In collaborations with more equiped research institutes in Western countries, plants and isolated compounds from the flora of CEWA were tested on cancer cells expressing well-known drug resistance phenotypes. In Tables 1, 2, results on samples are documented, that inhibited resistant cell lines with similar efficacy than sensitive ones (regular sensitivity). In some cases, it was observed that resistant cells were killed with even better efficacy than sensitive cells (hyper-sensitivity or collateral sensitivity). These plant extracts and phytochemicals could be especially useful to fight MDR in cancer. In this section, we will focus on plants and compounds exerting hypersensitivity on cell lines over-expressing ABC transporters, EGFR and with p53 knock out genes.

#### Plants and compounds acting in cancer cells over-expressing ABC transporters

Some botanicals and phytochemicals from CEWA were screened against ABC transporters-expressing cell lines. The most investigated cell lines included the P-gp-overexpressing CEM/ADR5000 leukemia cell line, the MRP1-expressing HL60/AR leukemia cell line and BCRP-expressing MDA-MB-231/*BCRP* breast adenocarcinoma cell line. The studies were mainly conducted by the team of Professor Thomas Efferth (University of Mainz, Germany). Plants and compounds inducing hypersensitivity in these cell lines are summarized in Tables 1, 2.

The hypersensitivity of CEM/ADR5000 cells compared to its parental cell line CCRF-CEM was induced by *Aframomum arundinaceum* (Oliver & Hanbury) K. Schum (Zingiberaceae) (Kuete et al., 2014a), *Imperata cylindrica* Beauv. var. koenigii Durand et Schinz (Poaceae) (Kuete et al., 2013c), *Nauclea pobeguinii* (Pobég. ex Pellegr.) Merr. ex E.M.A. (Rubiaceae) (Kuete et al., 2015f), *Pachypodanthium staudtii* Engl & Diels (Annonaceae) (Kuete et al., 2016b), *Piper capense* L.f. (Piperaceae) (Kuete et al., 2013c) and *Zinziber officinale Roscoe* (Zingiberaceae) (Kuete et al., 2011a). Compunds with similar activity included: acridone alkaloids, **49** and **53** (Kuete et al., 2015b), anthraquinone, **19** (Kuete et al., 2015a), flavonoids, 4′-hydroxy-2′,6′-dimethoxychalcone (**6**) from *Polygonum limbatum* Meisn (Kuete et al., 2014b), **12** (Kuete et al., 2016c), amentoflavone (**13**) from *Dorstenia barteri* (Kuete et al., 2016c), atalantoflavone (**15**) from *Erythrina sigmoidea* Hua (Kuete et al., 2014c), naringenin (**34**) from *Aframomum arundinaceum* (Oliver & Hanbury) K. Schum (Kuete et al., 2014a), lignans, futokadsurin B (**23**) from *Uapaca togoensis* Pax. (Kuete et al., 2016c), **39** and **40** (Kuete et al., 2016c) and xanthone **17** (Kuete et al., 2013b).

Plant extracts inducing hypersensitivity in MDA-MB-231-*BCRP* clone 23 cells compared to its sensitive counterparts MDA-MB-231 cells include: *Aframomum polyanthum* K. Schum (Zinziberaceae) (Kuete et al., 2014a), *Nauclea latifolia* Smith. (Rubiaceae) (Kuete et al., 2014a), *Nauclea pobeguinii* (Pobég. ex Pellegr.) Merr. ex E.M.A. (Kuete et al., 2015f), *Pachypodanthium staudtii* Engl & Diels (Kuete et al., 2016b) and *Uapaca togoensis* Pax. (Euphorbiaceae) (Kuete et al., 2015e) (Table 1). Compounds exerting similar activity included: alkaloids, **47, 48**, and **49** (Kuete et al., 2015b), diterpene, galanal A (**2**) isolated from *Aframomum arundinaceum* (Oliver & Hanbury) K. Schum (Kuete et al., 2014a), benzophenone, isogarcinol (**28**) from *Hypericum lanceolatum* Lam. (Kuete et al., 2013d), **30** (Kuete et al., 2013d), anthraquinones, **19** and **20** (Kuete et al., 2015a), flavonoids, **6** (Kuete et al., 2014b), **13** (Kuete et al., 2016c), **27** (Kuete et al., 2015c), kaempferol-3,7,4′-trimethylether (**31**) from *Aframomum arundinaceum* (Oliver & Hanbury) K. Schum (Kuete et al., 2014a), isoflavonoids, bidwillon A (**16**) from *Erythrina sigmoidea* Hua (Kuete et al., 2014c), **29** (Kuete et al., 2014d), **44** and **45** (Kuete et al., 2014c), lignan, **39** (Kuete et al., 2016c) and xanthones, 8-hydroxycudraxanthone G (**10**) from *Garcinia nobilis* Engl. (Kuete et al., 2013b), **17** (Kuete et al., 2013b) and euxanthone (**22**) (Kuete et al., 2016c).

#### Plants and compounds acting in EGFR over-expressing cancer cells

Several plants extracts and compounds were more active in the resistant gliobastoma U87MG.Δ*EGFR* cells than in its normal counterpart U87MG cells (D.R. < 0.90). They included: *Albizia adianthifolia* (Schum.) and *Alchornea cordifolia* (Schum. & Thonn.) Müll.-Arg. (Kuete et al., 2016e), *Anonidium mannii* Engl. et Diels. (Anonaceae) (Kuete et al., 2013a), *Elaeophorbia drupifera* (Thonn.) Stapf. (Euphorbiaceae) (Kuete et al., 2013e), *Erythrina sigmoidea* Hua (Kuete et al., 2014a), *Gladiolus quartinianus* A. Rich (Iridaceae) (Kuete et al., 2013a), *Nauclea pobeguinii* (Pobég. ex Pellegr.) Merr. ex E.M.A. (Kuete et al., 2015f), *Vepris soyauxii* Engl. (Rutaceae) (Kuete et al., 2013a) and *Xylopia aethiopica* (Dunal) A.Rich. (Annonaceae) (Kuete et al., 2013c). Compounds with similar activity include: the alkaloids, **47-53** (Kuete et al., 2015b,g), anthraquinone, **19** (Kuete et al., 2015a), benzophenones, 2,2′,5,6′-tetrahydroxybenzophenone (**3**) from *Hypericum lanceolatum* Lam. (Kuete et al., 2013d), **26** (Kuete et al., 2013d), **28** (Kuete et al., 2013d), **30** (Kuete et al., 2013d), flavonoids, **12** (Kuete et al., 2016c), dorsmanin F (**21**) (Kuete et al., 2015d), **27** (Kuete et al., 2015c), **34** (Kuete et al., 2014a), isoflavonoids, **16** (Kuete et al., 2014c), **35** (Kuete et al., 2014d), **44** and **45** (Kuete et al., 2014c), xanthones, **10** (Kuete et al., 2013b), and **17** (Kuete et al., 2013b).

#### Plants and compounds acting in p53 knockout cancer cells

Botanicals inducing hypersensitivity in p53 knockout cell line HCT116 (*p53*^−/−^) compared to its sensitive counterpart HCT116 (*p53*^+/+^) cell line included: *Beilschmiedia acuta* Kosterm (Lauraceae) (Kuete et al., 2014e), *Echinops giganteus* var. lelyi (C. D. Adams) A. Rich. (Compositae) (Kuete et al., 2013c), *Erythrina sigmoidea* Hua (Fabaceae) (Kuete et al., 2014a), *Nauclea latifolia* Smith. (Kuete et al., 2014a), *Nauclea pobeguinii* (Pobég. ex Pellegr.) Merr. ex E.M.A. (Kuete et al., 2015f), *Polyscias fulva* (Hiern) Harms. (Araliaceae) (Kuete et al., 2014e) and *Uapaca togoensis* Pax. (Kuete et al., 2015e). Compounds acting in p53 knockout cancer cells included: alkaloid, **48** (Kuete et al., 2015b), benzophenone, **26** (Kuete et al., 2013d), flavonoids, **12** (Kuete et al., 2016c), **27** (Kuete et al., 2015c), laburnetin (**32**) (Kuete et al., 2016c), **38** (Kuete et al., 2015d), isoflavonoids, **9** (Kuete et al., 2014c), **11** (Kuete et al., 2014c), **16** (Kuete et al., 2014c), **29** (Kuete et al., 2014d), **44** (Kuete et al., 2014c), neobavaisoflavone (**35**) from *Erythrina senegalensis* DC (Kuete et al., 2014d), lignan, **23** (Kuete et al., 2016c), xanthones, **10** (Kuete et al., 2013b), and **22** (Kuete et al., 2016c).

## Hit cytotoxic plants of central, east and west africa

Some African plant extracts displayed very interresting cytotoxic effects with IC_50_ values below 20 μg/mL in the majority of cancer cell lines tested. In this section, the synopsis of 10 strongest cytotoxic plants of CEWA as observed with *in vitro* screening assays is provided.

### *Beilschmiedia acuta* kosterm (lauraceae)

*Beilschmiedia acuta* [Synonyms: *Beilschmiedia acutifolia* (Engl. & K. Krause) Robyns & Wilczek or *Tylostemon acutifolius* Engl. & K. Krause] belongs to the family Lauraceae. The plant is mainly found in Cameroon and Central African Republic, where it is traditionally used to treat cancer and gastrointestinal infections (Kuete et al., 2014e). The methanol extracts of leaves and roots of the plant were tested on a panel of cancer cell lines, including MDR phenotypes. Both leaves and roots extracts displayed good antiproliferative effects with respective IC_50_ values of 8.22 and 14.72 μg/mL in leukemia CCRF-CEM cells, 19.76 and 26.74 μg/mL in its resistant subline CEM/ADR5000 cells, 6.45 and 6.66 μg/mL in breast adenocarcinoma MDA-MB-231 cells and 21.09 and 22.75 μg/mL in its resistant counterparts MDA-MB-231/*BCRP*, 21.12 and 11.62 μg/mL in colon adenocarcinoma HCT116 *p53*^+/+^and its resistant counterparts HCT116 *p53*^−/^^−^, 7.46 and 7.27 μg/mL in gliobastoma U87MG cells and its resistant counterparts U87MG.Δ*EGFR* cells and 23.09 μg/mL for leaves extract in HepG2 cells (Kuete et al., 2014e). Interestingly, the two extracts were less toxic toward normal AML12 hepatocytes with IC_50_ values above 40 μg/mL (Kuete et al., 2014e). Both leaves and roots extracts induced apoptosis in CCRF-CEM cells. However, the mode of induction of apoptosis was not dectected when MMP and ROS production were investigated (Kuete et al., 2014e).

### *Echinops giganteus* var. Lelyi (C. D. Adams) A. Rich. (composiatae)

*Echinops giganteus* is a medicinal spicy plant of the family Compositae mainly found in Cameroon, Ethiopia, Rwanda, Sudan, Tanzania, Uganda, DR Congo. The plant is traditionally used to treat cancer, as well as heart and gastric troubles (Tene et al., 2004; Kuete et al., 2011a). The methanol extract of the rhizomes of the plant displayed good antiproliferative effects toward leukemia CCRF-CEM cells (IC_50_: 6.68μg/mL), CEM/ADR5000 cells (IC_50_: 7.96 μg/mL) (Kuete et al., 2011a), HL60 cells (IC_50_: 6.38 μg/mL) and HL60AR cells (IC_50_: 9.24 μg/mL), MDA-MB-231-*pcDNA* cells (IC_50_: 8.61 μg/mL), MDA-MB-231-*BCRP* cells (IC_50_: 6.52 μg/mL), colon carcinoma HCT116 (*p53*^+/+^) cells (IC_50_: 3.58 μg/mL), HCT116 (*p53*^−/−^) cells (IC_50_: 3.29 μg/mL), gliobastoma U87MG cells (IC_50_: 13.55 μg/mL) and U87MG.Δ*EGFR* cells (IC_50_: 11.15 μg/mL), hepatocarcinoma HepG2 cells (IC_50_: 14.32 μg/mL) (Kuete et al., 2013c). Importantly, this extract was less toxic to the normal human umbilical vein endothelial cells (HUVECs; IC_50_> 80 μg/mL) (Kuete et al., 2011a) and to normal AML12 hepatocytes with less than 50% proliferation at 40 μg/mL (Kuete et al., 2013c). This extract induced apoptosis in CCRF-CEM cells by loss of MMP (Kuete et al., 2013c).

### Erythrina sigmoidea hua (fabaceae)

*Erythrina sigmoidea* (synonyms: *Erythrina dybowskii* Hua; *Erythrinaeriotricha* Harms; *Erythrina lanata* Taub. ex Gilg; *Erythrina sudanica* Baker f.) is a tree of 3–6 m, or 10–20 m belonging to the Fabaceae family. The plant is mainly found in Cameroon and Chad, where it is used as antidote (venomous stings, bites, etc.), diuretic, febrifuge and to treat arthritis, rheumatism, pulmonary troubles, stomach troubles, infectious diseases and kidney diseases (Burkill, 1985), gastrointestinal infections, venereal diseases and leprosy (Mabeku et al., 2011). The cytotoxic constituents of the plant include 6α-hydroxyphaseollidin (**9**), atalantoflavone (**15**), bidwillon A (**16**), neobavaisoflavone (**35**), neocyclomorusin (**36**), and sigmoidin I (**44**) (Kuete et al., 2014c). The cytotoxicity of bark methanol extract was reported toward CCRF-CEM cells (IC_50_: 18.50 μg/mL), CEM/ADR5000 cells (IC_50_: 20.06 μg/mL), MDA-MB-231-*pcDNA* cells (IC_50_: 22.37 μg/mL), MDA-MB-231-*BCRP* cells (IC_50_: 27.42 μg/mL), HCT116 (*p53*^+/+^) cells (IC_50_: 19.63 μg/mL), HCT116 (*p53*^−/−^) cells (IC_50_: 16.22 μg/mL), U87MG cells (IC_50_: 45 μg/mL), U87MG.Δ*EGFR* cells (IC_50_: 29.80 μg/mL), and HepG2 cells (IC_50_:22.34 μg/mL) (Kuete et al., 2016a). This extract had low cytotoxicity toward normal AML12 hepatocytes, inducing less than 50% proliferation at 80 μg/mL (Kuete et al., 2016a). It induced apoptosis in CCRF-CEM leukemia cells by disruption of the MMP (Kuete et al., 2014a).

### *Imperata cylindrica* beauv. var. koenigii durand et schinz (poaceae)

*Imperata cylindrica* commonly known as cogon grass is a perennial rhizomatous grass of the Poaceae family. The plant is native to East and south East Asia, India, Micronesia, Melanesia, Australia, and Eastern and Southern Africa. In CEWA, the plant is found in Benin, Burkina Faso, Congo, Ivory Cost, Gambia, Ghana, Guinea, Kenya, Liberia, Mali, Mozambique, Niger, Nigeria, Senegal, Tanzania, Togo, Uganda. The plant is traditionally used as diuretic and anti-inflammatory agents and to treat cancer (Nishimoto et al., 1968; Kuete et al., 2011a). The cytotoxicity of roots methanol extract of the plant was reported toward CCRF-CEM cells (IC_50_: 8.4 μg/mL) and CEM/ADR5000 cells (IC_50_: 7.18 μg/mL), pancreatic MiaPaca-2cells (IC_50_: 12.11 μg/mL) (Kuete et al., 2011a), HL60 cells (IC_50_: 7.94 μg/mL), HL60AR cells (IC_50_: 30.60 μg/mL), MDA-MB-231-*pcDNA* cells (IC_50_: 5.19 μg/mL), MDA-MB-231-*BCRP* cells IC_50_: 10.04 μg/mL), HCT116 (*p53*^+/+^) cells (IC_50_: 4.37 μg/mL), HCT116 (*p53*^−/−^) cells (IC_50_: 4.60 μg/mL), U87MG cells (IC_50_: 19.99 μg/mL), U87MG.Δ*EGFR* cells (IC_50_: 10.68 μg/mL), and HepG2 cells (IC_50_: 18.28 μg/mL) (Kuete et al., 2013c). Less than 50% proliferation of CCRF-CEM cells was induced by this extract in normal AML12 hepatocytes (Kuete et al., 2013c) meanwhile the IC_50_ value as high as 47.73 μg/mL was obtained in HUVEC cells. This extract induced apoptosis in CCRF-CEM cells by loss of MMP (Kuete et al., 2013c).

### *Nauclea pobeguinii* (pobég. ex pellegr.) merr. ex E.M.A. (rubiaceae)

*Nauclea pobeguinii* (synonym: *Sarcocephalus pobeguinii* Pobég. ex Pellegr.) is a deciduous, small to medium-sized tree growing up to 30 m tall, sometimes a shrub. In CEWA, the plant is distributed in South Tropical Africa especially in Angola, Zambia, West Tropical Africa: Burkina, Ghana, Guinea, Guinea-Bissau, Ivory Coast, Nigeria, Senegal, Sierra Leone, West-Central Tropical Africa: Cameroon, Central African Republic, Congo, DR Congo, Gabon. The plant is used in traditional medicine as abortive and to treat stomach ache and infectious diseases (Karou et al., 2011), jaundice (Kadiri et al., 2007), fever, diarrhea, worm, and malaria (Mesia et al., 2005). The cytotoxicity of the methanol extract from bark and leaves was reported toward CCRF-CEM cells (IC_50_: 14.62 and 25.84 μg/mL, respectively), CEM/ADR5000 cells (IC_50_: 11.56 and 25.55 μg/mL, respectively), HCT116 (*p53*^+/+^) cells (IC_50_: 16.19 and 32.72 μg/mL, respectively) and HCT116 (*p53*^−/−^) cells (IC_50_: 8.70 and 19.39 μg/mL, respectively) (Kuete et al., 2015f). Resveratrol was identified as the major cytotoxic constituent of this extract (Kuete et al., 2015f).

### *Piper capense* L.f. (piperaceae)

*Piper capense* is a rather variable spicy plant ranging from a weakly erect, aromatic, evergreen shrub or subshrub, to a more or less herbaceous perennial and sometimes a straggling plant that scrambles into other plants for support. *Piper capense* is found from Guinea to Ethiopia, Angola and Mozambique. Traditionally, the plant is used as sleep inducing remedy, anthelmintic and to treat cancer (Kokowaro, 1976; Van Wyk and Gericke, 2000; Kuete et al., 2011a). The cytotoxicity of seeds methanol extract was reported toward CCRF-CEM cells (IC_50_: 7.03 μg/mL), CEM/ADR5000 (IC_50_: 6.56 μg/mL) and MiaPaca-2 cells (IC_50_: 8.92 μg/mL) (Kuete et al., 2011a), HL60 cells (IC_50_: μg/mL), HL60AR cells (IC_50_: μg/mL), MDA-MB-231-*pcDNA* cells (IC_50_:4.17 μg/mL), MDA-MB-231-*BCRP* cells (IC_50_: 19.45 μg/mL), HCT116 (*p53*^+/+^) cells (IC_50_: 4.64 μg/mL), HCT116 (*p53*^−/−^) cells (IC_50_: 4.62 μg/mL), U87MG cells (IC_50_: 13.48 μg/mL), U87MG.Δ*EGFR* cells (IC_50_: 7.44 μg/mL), HepG2 cells (IC_50_: 16.07 μg/mL) (Kuete et al., 2013c). This extract was less toxic toward normal AML12 hepatocytes and HUVEC cells inducing less than 50% cell proliferation at 40 μg/mL and 80 μg/mL respectively (Kuete et al., 2011a, 2013c). This extract induced apoptosis in CCRF-CEM cells by the loss of MMP and increase ROS production (Kuete et al., 2013c).

### *Polyscias fulva* (hiern) harms. (araliaceae)

*Polyscias fulva* is a deciduous to evergreen tree of the family Araliaceae. The plant is found in Tropical Africa, from Sierra Leone to Sudan, Ethiopia, and Yemen; in Angola, Zambia, Zimbabwe, and Mozambique. Traditionally, *Polyscias fulva* is used to treat malaria, fever, mental illness (Tshibangu et al., 2002), venereal infections and obesity (Jeruto et al., 2007; Focho et al., 2009), and cancer (Kuete et al., 2014e). The phytochemical investigations of the plant led to the isolation of polysciasoside A, kalopanax-saponin B, α-hederin (Bedir et al., 2001; Kuete and Efferth, 2011). Investigation of the cytotoxic potential of various parts of the plant demonstrated that the roots were more active than the leaves and bark (Kuete et al., 2014e). Roots methanol extract had good cytotoxic effects on a panel of human cancer cell lines with the IC_50_ values of 7.79 μg/mL (CCRF-CEM cells), 22.63 μg/mL (CEM/ADR5000 cells), 3.27 μg/mL (MDA-MB-231 cells), 16.67 μg/mL (MDA-MB-231/*BCRP* cells), 14.66 μg/mL (HCT116 *p53*^+/+^cells), 5.98 μg/mL (HCT116 *p53*^−/^^−^cells), 4.15 μg/mL (U87MG cells), 16.35 μg/mL (U87MG.Δ*EGFR* cells), and 12.99 μg/mL (HepG2 cells) (Kuete et al., 2014e). Lower cytotoxicity of this extract was shown in normal AML12 hepatocytes with less than 50% cells proliferation at 40 μg/mL (Kuete et al., 2014e). The active constituent of the plant was reported as α-hederin and this compound had moderate antiproliferative effects (IC_50_ values ranged from 7.43 μM in CCRF-CEM cells to 43.98 μM in U87MG.Δ*EGFR* cells) against the above cancer cell lines (Kuete et al., 2014e). The roots methanol extract of *Polyscias fulva* induced apoptosis in CCRF-CEM cells, mediated by MMP alterations and increased ROS production (Kuete et al., 2014e).

### *Uapaca togoensis* pax (euphorbiaceae)

*Uapaca togoensis* (Synonyms:*Uapaca chevalieri* Beille, *Uapaca guignardii* A.Chec. ex Beille, *Uapaca guineensis* sudanica (Beille) Hutch., *Uapaca perrotii* Beille *Uapaca somon* Aubrév. & Leandri) is an evergreen tree. The plant grows in tropical Africa, from Senegal to southern Chad and Central African Republic and from Gabon to DR Congo and northern Angola. In traditional medicine, the plant is used as antiemetic, lotion for skin disorders (Mengome et al., 2010), remedy for pneumonia, cough, fever, rheumatism, vomiting, epilepsy (Kone et al., 2006) and bacterial diseases (Kone et al., 2004). The cytotoxicity of the methanol extract from fruit was reported toward CCRF-CEM cells (IC_50_: 4.23 μg/mL), CEM/ADR5000 cells (IC_50_: 4.44 μg/mL), MDA-MB-231-*pcDNA* cells (IC_50_: 25.85 μg/mL), MDA-MB-231-*BCRP* cells (IC_50_: 4.17 μg/mL), HCT116 (*p53*^+/+^) cells (IC_50_: 3,69 μg/mL), HCT116 (*p53*^−/−^) cells (IC_50_: 3.09 μg/mL), U87MG cells (IC_50_: 8.01 μg/mL), U87MG.Δ*EGFR* cells (IC_50_: 8.68 μg/mL), and HepG2 cells (IC_50_: 19.90 μg/mL) (Kuete et al., 2015e). This extract induced apoptosis in CCRF-CEM cells mediated by MMP loss (Kuete et al., 2015e). The cytotoxic consitutuents of the extract were identified as a terpenoid, 11-oxo-α-amyryl acetate, a lignan, futokadsurin B (**23**) and an alkaloid,arborinin (**49**) (Kuete et al., 2015e).

### *Vepris soyauxii* engl. (rutaceae)

*Vepris soyauxii* (synonym: *Araliopsis soyauxii* Engl) is a plant of the family Rutaceae mostly found throughout West Africa, from Sierra Leone, Liberia, Ivory Cost, Mali, Ghana to Nigeria, and Cameroon. In traditional medicine, the plant is used as anti-fibriomyoma and to treat stomachache, malaria (Momeni et al., 2010) and cancer (Kuete et al., 2013a). The antiproliferative effects of the methanol extract from leaves was reported toward CCRF-CEM cells (IC_50_: 9.28 μg/mL), CEM/ADR5000 cells (IC_50_: 11.72 μg/mL), MDA-MB-231-*pcDNA* cells (IC_50_: 7.52 μg/mL), MDA-MB-231-*BCRP* cells (IC_50_: 12.93 μg/mL), HCT116 (*p53*^+/+^) cells (IC_50_: 8.59 μg/mL), HCT116 (*p53*^−/−^) cells (IC_50_:9.70 μg/mL), U87MG cells (IC_50_: 8.75 μg/mL), U87MG.Δ*EGFR* cells (IC_50_: 4.09 μg/mL) and HepG2 cells (IC_50_: 13.60 μg/mL) (Kuete et al., 2013a). This extract induced apoptosis in CCRF-CEM cells mediated by disruption of MMP (Kuete et al., 2013a). Besides, this extract had low cytotoxic effect toward normal AML12 hepacytes, inducing less than 50% proliferation at 40 μg/mL (Kuete et al., 2013a).

### *Xylopia aethiopica* (dunal) A. rich. (annonaceae)

*Xylopia aethiopica* is an aromatic tree of the family Annonaceae. The plant is native to the lowland rainforest and moist fringe forests in the savanna zones of Africa. The plant grows in Angola, Benin, Burkina Faso, Cameroon, Central African Republic, DR Congo, Ethiopia, Gabon, Gambia, Ghana, Guinea, Guinea-Bissau, Ivory Coast, Kenya, Liberia, Mozambique, Nigeria, São Tomé and Príncipe, Senegal, Sierra Leone, Sudan, South Sudan, Tanzania, Togo, and Uganda. Traditionally, this plant is used to treat cancer, constipation, uterine hemorrhage, fever and as diuretic (Iwu, 1993; Kuete et al., 2011a; Okafor, 2012). The cytotoxicity of seeds methanol extract was demonstrated toward CCRF-CEM cells (IC_50_: 3.91 μg/mL), CEM/ADR5000 cells (IC_50_:7.4 μg/mL)and Mia PaCa-2 cells (IC_50_: 6.86 μg/mL) (Kuete et al., 2011a), human cervical cancer cell line C-33A (IC_50_:30.8μg/mL), breast adonocarcinoma MCF7 cells (IC_50_: 60.2 μg/mL), human oral squamous carcinoma KB cells (IC_50_: 62.5 μg/mL) (Adaramoye et al., 2011), HL60 cells (IC_50_: 7.94 μg/mL), HL60AR cells (IC_50_: 30.60 μg/mL), MDA-MB-231-*pcDNA* cells (IC_50_: 5.19 μg/mL), MDA-MB-231-*BCRP* cells (IC_50_: 10.04 μg/mL), HCT116 (*p53*^+/+^) cells (IC_50_: 4.37 μg/mL), HCT116 (*p53*^−/−^) cells (IC_50_: 4.60 μg/mL), U87MG cells (IC_50_: 19.99 μg/mL), U87MG.Δ*EGFR* cells (IC_50_: 10.68 μg/mL) and HepG2 cells (IC_50_: 18.28 μg/mL) (Kuete et al., 2013c). This extract was less toxic against AML12 hepatocytes and HUVEC cells inducing less than 50% cell proliferation at 40 and 80 μg/mL, respectively (Kuete et al., 2011a, 2013c). Its also induced apoptosis in C-33A cells, nuclear fragmentation, cells accumulation in sub-G0/G1, cycle arrest in G2, up-regulation of p53 and p21 genes, and an increase in the Bax/Bcl-2 ratio (Adaramoye et al., 2011). It also induced apoptosis in CCRF-CEM cells by loss of MMP (Kuete et al., 2013c). The cytotoxic constituents of this extract were identified as 16α-hydroxy-ent-kauran-19-oic acid, 3,4′,5-trihydroxy-6″,6″-dimethylpyrano[2,3-g]flavone, isotetrandrine (**51**) and *trans*-tiliroside (Kuete et al., 2015g).

## Hit cytotoxic compounds from plants of central, eastern and western africa

Several bioactive consituents of African medicinal plants were identified. They include: terpenoids, phenolics and alkaloids (Table 2). However, phenolics were the best cytotoxic ingredients isolated from CEWA plants. In this section, a summary of the prominent antiproliferative phytochemicals identified in CEWA plant will be given.

### Alkaloids

The isoquinoline alkaloid, isotetrandrine (**51**) isolated from *Xylopia aethiopica* was amongst the most active alkaloids reported in CEWA plants. This compound displayed interesting cytotoxic effects with IC_50_ values below 10 μM toward a panel of sensitive and MDR cancer cell lines. These cell lines included: CCRF-CEM cells (IC_50_: 1.53 μM), CEM/ADR5000 cells (IC_50_: 2.36 μM), MDA-MB-231-*pcDNA* cells (IC_50_: 7.28 μM), MDA-MB-231-*BCRP* cells IC_50_:6.70 μM), HCT116 (*p53*^+/+^) cells (IC_50_: 2.39 μM), HCT116 (*p53*^−/−^) cells(IC_50_: 4.55 μM), U87MG cells (IC_50_: 3.89 μM), U87MG.Δ*EGFR* cells (IC_50_: 1.45 μM) and HepG2 cells (IC_50_: 3.28 μM) (Kuete et al., 2015g). Alkaloid, **51** was less toxic against the normal AML12 hepatocytes, inducing less than 50% proliferation at up to 64.27 μM (Kuete et al., 2015g). This compound did not alter the integrity of the mitochondrial membrane in CCRF-CEM cells, and its mode of induction of apoptosis was mainly by increased ROS production (Kuete et al., 2015g).

### Phenolic compounds

Phenolics have been so far the most represented group of secondary metabolites isolated from CEWA medicinal plants. Several compounds with interesting cytotoxic activities were identified within benzophenones, flavonoids and isoflavonoids, naphthyl butenone, quinones and xanthones.

### Benzophenones

Guttiferone E (**26**) and isoxanthochymol (**30**) isolated from *Garcinia punctata Oliv*. (Guttiferae) (Kuete et al., 2013d) showed good cytotoxic effects against a panel of human cancer cell lines. Compounds **26** and **30** have also been isolated from various *Garcinia species* such as *Garcinia pyrifera* (Roux et al., 2000), *Garcinia xanthochymus* (Baggett et al., 2005), *Garcinia virgata* (Merza et al., 2006), *Garcinia afzelii* (Lannang et al., 2010), *Garcinia livingstonei* (Yang et al., 2010), *Garcinia multiflora* (Liu et al., 2010) and from *Rheedia edulis* (Acuna et al., 2010). Compound **26** had an IC_50_ value of 7.5 μM toward colon carcinoma SW-480 cells, meanwhile **30** was less active in this cell line (IC_50_: 16.6 μM) (Baggett et al., 2005). Benzophenones **26** and **30** displayed good cytotoxic effects toward CCRF-CEM cells (IC_50_: 6.86 and 9.55 μM, respectively), CEM/ADR5000 cells(IC_50_:13.57 and 10.33 μM, respectively), HL60 cells (IC_50_: 11.69 and 8.92 μM, respectively), HL60AR cells (IC_50_: 11.69 and 8.92 μM, respectively), MDA-MB-231-*pcDNA* cells (IC_50_:11.69 and 6.30 μM, respectively), MDA-MB-231-*BCRP* cells (IC_50_: 13.92 and 3.42 μM, respectively), HCT116 (*p53*^+/+^) cells (IC_50_:12.74 and 3.24 μM, respectively), HCT116 (*p53*^−/−^) cells (IC_50_: 7.87 and 3.62 μM, respectively), U87MG cells (IC_50_: 7.87 and 6.40 μM, respectively), U87MG.Δ*EGFR* cells (IC_50_: 3.39 and 3.12 μM, respectively) and HepG2 cells (IC_50_: 11.13 and 8.34 μM, respectively) (Kuete et al., 2013d). Both **26** and **30** induced apoptosis in cervix adenocarcinoma HeLa cells (Liu et al., 2010). The two compounds also induced apoptosis in CCRF-CEM cells by activation of caspases 3/7, 8 and 9 and loss of MMP (Kuete et al., 2013d).

### Flavonoids and isoflavonoids

Flavonoids and isoflavonoids are amongst the most isolated and the most active phytochemicals identified in African medicinal plants (Kuete and Efferth, 2015). Well studied flavonoids from CEWA plants having prominent cytotoxic effect against human cancer cell lines include 4′-hydroxy-2′,6′-dimethoxychalcone (**6**), isobavachalcone (**27**), neocyclomorusin (**36**), poinsettifolin B (**38**), 6α-hydroxyphaseollidin (**9**), isoneorautenol (**29**), neobavaisoflavone (**35**), sigmoidin I (**44**) and sophorapterocarpan A (**45**) (Table 2). Amongst them, isoflavonoid **9** revealed the best activity with IC_50_ values below 10 μM on a panel of cancer cell lines including CCRF-CEM cells (IC_50_: 3.36 μM), CEM/ADR5000 cells (IC_50_: 5.51 μM), MDA-MB-231-*pcDNA* cells (IC_50_: 5.70 μM), MDA-MB-231-*BCRP* cells (IC_50_: 5.87 μM), HCT116 (*p53*^+/+^) cells (IC_50_: 5.68 μM), HCT116 (*p53*^−/−^) cells (IC_50_: 4.60 μM), U87MG cells (IC_50_: 4.91 μM), U87MG.Δ*EGFR* cells (IC_50_: 4.91 μM) and HepG2 cells (IC_50_: 6.44 μM)(Kuete et al., 2014c). Compound **9** induced apoptosis in CCRF-CEM cells by the activation of caspases 3/7, 8 and 9 and breakdown of MMP as well as increased ROS production (Kuete et al., 2014c).

### Naphthyl butenone

Guieranone A (**25**), a major component of the leaves of *Guiera senegalensis* displayed good cytotoxic effects on a panel of human cancer cell lines. The cytotoxicity of **25** was documented toward CCRF-CEM cells (IC_50_: 2.31 μM), CEM/ADR5000 cells (IC_50_: 3.19 μM), MiaPaCa-2 cells (IC_50_: 12.39 μM), Capan-1 cells (IC_50_: 29.08 μM), MCF-7 cells (IC_50_: 3.42μM), 786-0 cells (IC_50_: 11.32 μM), U87MG cells (IC_50_: 7.78 μM), lung carcinoma A549 cells (IC_50_: 2.28 μM), skin melanoma Colo-38 cells (IC_50_: 7.69 μM), cervical carcinoma HeLa cells (IC_50_: 1.61 μM), and Caski cells (IC_50_: 3.73 μM) (Kuete et al., 2012) and leukemia THP-1 cells (IC_50_: 13.43 μM) (Fiot et al., 2006). Compound **25** showed anti-angiogenic activity *via* the inhibition of the growth of blood capillaries on the chorioallantoic membrane of quail embryo. It also induces apoptosis in CCRF-CEM cells. Meanwhile, microarray analysis demonstrated that it affected the regulation of several pathways in CCRF-CEM cells, including the cell cycle: G2/M DNA damage checkpoint regulation and ATM signaling pathways (Kuete et al., 2012).

### Quinones

Two naphthoquinones isolated from African plants, 2-acetylfuro-1,4-naphthoquinone (**4**) and plumbagin (**37**), showed remarkable cytotoxic effects (Table 2). Compound **4** showed good cytotoxicity toward PF-382 cells (IC_50_: 2.36 μM), MiaPaCa-2 cells (IC_50_: 7.48 μM), MCF-7 cells (IC_50_: 6.68 μM), U87MG cells (IC_50_: 8.02 μM), Colo-38 cells (IC_50_: 2.77 μM), HeLa cells (IC_50_: 1.65 μM), and Caski cells (IC_50_: 0.70 μM) (Kuete et al., 2011d). Naphthoquinone **4** revealed anti-angiogenic effects through inhibition of the growth of blood capillaries on the chorioallantoic membrane of quail eggs and also induced apoptosis and cell cycle arrest in S-phase in CCRF-CEM (Kuete et al., 2011d). The cytotoxic potential of compound **37** has widely been reported (Srinivas et al., 2004; Kuo et al., 2006; Powolny and Singh, 2008; Sun and McKallip, 2011; Kawiak et al., 2012). It has been isolated from various species of Plumbaginaceae, Ebenaceae and Droseraceae (Sagar et al., 2014). Compound **37** induced ROS mediated apoptosis in human cervical cancer ME-180 cells (Srinivas et al., 2004), human prostate cancer PC-3 cells and LNCaP cells (Powolny and Singh, 2008), G2-M arrest and autophagy by inhibiting the AKT/mammalian target of rapamycin (mTOR) pathway in MCF-7 cells and MDA-MD-231 cells (Kuo et al., 2006). Naphthoquinone **37** also induced apoptosis in Her2-overexpressing breast cancer cells through the mitochondrial-mediated pathway (Kawiak et al., 2012) as well as in human K562 leukemia cells through increased ROS and elevated tumor-necrosis-factor related apoptosis inducing ligand (TRAIL) receptor expression (Sun and McKallip, 2011).

### Xanthones

Xanthone V_1_ (**46**) (Table 2) is one of the most active xanthones with prominent cytotoxic effects isolated from CEWA plants, displaying IC_50_ values below or around 10 μM against MCF-7 cells (IC_50_: 1.42 μM), 786-0 cells (IC_50_: 9.62 μM), U87MG cells (IC_50_: 9.64 μM), A549 cells (IC_50_:10.13 μM), Colo-38 cells (IC_50_: 3.02 μM), HeLa cells (IC_50_: 0.58 μM), and Caski cells (IC_50_: 0.61 μM) (Kuete et al., 2011d). This compound had anti-angiogenic effects, inhibiting the growth of blood capillaries on the chorioallantoic membrane of quail eggs. Compound **46** induced apoptosis and cell cycle arrest in S-phase in CCRF-CEM cells mediated by caspase 3/7 activation (Kuete et al., 2011d).

## Conclusion

The present review paper aimed at compiling and summarizing relevant data on the potential of medicinal plant and isolated natural products from Central, Eastern and Western Africa to combat cancer with emphasis on their possible cellular targets. This report could not deliver medical results on the therapeutic capacities of the flora of these three African Regions as anticancer drugs. Nonetheless, in phytochemical and pharmacological basic sciences it clearly shows that efforts are being made by African scientists and their international collaborators to achieve this goal in the future. However, few research teams in the continent are already involved in the cytotoxic drug discovery from botanicals and it is expected that this review will stimulate other laboratories to undertake similar research projects to better valorize the African flora.

## Author contributions

AM and VK wrote the manuscript; VK and TE designed and corrected the work. All authors read and approved the final version.

### Conflict of interest statement

The authors declare that the research was conducted in the absence of any commercial or financial relationships that could be construed as a potential conflict of interest.

## Abbreviations

4E-BP1: 4E-binding protein 1
ABC transporters: ATP binding cassette transporters
Akt: protein kinase B
AMPK: AMP-activated protein kinase
Apaf-1: apoptotic peptidase activating factor 1
AR: androgen receptor
ARE: antioxidant response element
ATM/Chk1: ataxia–telangiectasia-mutated/check point kinase-1
BAD: Bcl-2-associated agonist of cell death
Bax: Bcl-2-associated X protein
BCRP: breast cancer resistance protein
BRCA: breast cancer
BTG3: B-cell translocation gene 3
C/EBPα: CAAT-enhancer binding protein α;
CCL2: CC motif ligand 2
Cdc2: cyclin-dependent kinase 1
Cdc25a: cell division cycle 25 homolog A
cIAP: cellular inhibitor of apoptosis protein
COX: cyclooxygenase
CXCL: chemokine (C-X-C motif) ligand
CXCR4: chemokine (C-X-C motif) receptor 4
CYP1A1: cytochrome P450 1A1
DR5: death receptor 5
EGFR: epidermal growth factor receptor
EMMPRIN: extracellular matrix metalloproteinase inducer
ErbB2: Receptor tyrosine-protein kinase
ERK: extracellular signal-regulated kinase
ER-α, -β: estrogen receptor -α, -β
Fas: Fatty acid synthase
FoxM1: forkhead box M1
FOXO: forkhead transcription-factor O
G3BP1: GTPaseactivating protein (SH3 domain) binding protein 1
GDF15: growth differentiation factor 15
Gli1: glioma-associated oncogene 1
GST: glutathione transferase
HIF-1α: hypoxia inducible factor 1, alpha
HMG-CoA: hydroxy-methylglutaryl-coenzyme A
Hsp70: heat shock 70 kDa protein
hSULT1A1: human simple phenol sulfotransferase
hSULT2A1: human dehydroepiandrosterone sulfotransferase
IDO: indole amine 2,3-dioxygenase
IGF-1R: insulin-like growth factor -1 receptor
IGFBP: insulin-like growth factor binding protein
IGF: insulin-like growth factor
IKK: Ikappa beta kinase
IL: interleukin
IL-1RI: IL-1 receptor type I
iNOS: inducible nitric oxide synthase
JNK: c-Jun NH(2)-terminal kinase
MAPK: mitogen activated protein kinase
MDR: multi-drug resistance
MMP-2, -7, -9: matrix metallopeptidase-2, -7, -9
MMP: mitochondrial membrane potential
MRPs: multi drug resistance proteins
mTOR: mammalian target of rapamycin
NF-κB: nuclear factor-kappaB
Nrf2: nuclearfactor erythroid 2 related factor 2
PAK1: p21-activated protein kinase 1
PFKFB4: 6-phosphofructo-2-kinase/fructose-2,6-biphosphatase 4
PGE2: prostaglandin-E2;P-gp, P-glycoprotein
PHB: prohibitin
PI3K: phosphatidylinositol 3-kinase
Pin1: peptidyl prolyl cis/trans isomerase
PKC-α, -δ: protein kinase C -α, -δ
PPARγ: peroxisome proliferator-activated receptor-gamma
PTEN: phosphatase and tensin homolog
QR: quinone reductase
RAR-α, -β: retinoic acid receptor-α, -β
RB: retinoblastoma
Skp2: S-phase kinase-associated protein 2
SphK1: sphingosine kinase 1
TGFβ: Transforming growth factor-β
TNF: tumor necrosis factor
TRAIL: TNF-related *apoptosis*-inducing ligand
UGT1A: uridine 5′-diphosphate-glucuronosyltransferase 1A
VDR: vitamin D receptor
VEGF: vascular endothelial growth factor
VEGFR: VEGF receptor
WIF-1: Wnt inhibitory factor-1
XIAP: X-linked inhibitor of apoptosis
ZEB1: zinc finger E-box binding homeobox 1.
